# Research on the Digital Twin System of Welding Robots Driven by Data

**DOI:** 10.3390/s25133889

**Published:** 2025-06-22

**Authors:** Saishuang Wang, Yufeng Jiao, Lijun Wang, Wenjie Wang, Xiao Ma, Qiang Xu, Zhongyu Lu

**Affiliations:** 1 College of Electrical Engineeringy, North China University of Water Resources and Electric Power, Zhengzhou 450045, China; wangsaishuang@ncwu.edu.cn; 2 Management Center of Xiaolangdi Water Conservancy Hub of Ministry of Water Resources, Jiyuan 459000, China; jiaoyufeng@xiaolangdi.com.cn; 3 School of Mechanical Engineering, North China University of Water Resources and Electric Power, Zhengzhou 450045, China; wangwenjie23333@163.com; 4 School of Computing and Engineering, University of Huddersfield, Huddersfield HD1 3DH, UK; q.xu2@hud.ac.uk; 5 The Glass Box, 6 Friendly Street, Huddersfield, HD1 1RD, UK

**Keywords:** Welding robotic arm, 3D modeling, Digital twin, virtual simulation

## Abstract

With the rise of digital twin technology, the application of digital twin technology to industrial automation provides a new direction for the digital transformation of the global smart manufacturing industry. In order to further improve production efficiency, as well as realize enterprise digital empowerment, this paper takes a welding robot arm as the research object and constructs a welding robot arm digital twin system. Using three-dimensional modeling technology and model rendering, the welding robot arm digital twin simulation environment was built. Parent–child hierarchy and particle effects were used to truly restore the movement characteristics of the robot arm and the welding effect, with the help of TCP communication and Bluetooth communication to realize data transmission between the virtual segment and the physical end. A variety of UI components were used to design the human–machine interaction interface of the digital twin system, ultimately realizing the data-driven digital twin system. Finally, according to the digital twin maturity model constructed by Prof. Tao Fei’s team, the system was scored using five dimensions and 19 evaluation factors. After testing the system, we found that the combination of digital twin technology and automation is feasible and achieves the expected results.

## 1. Introduction

With the rapid development of digital communication technology, a new round of industrial transformation has been unfolding globally, with intelligent manufacturing supported by cloud computing, big data, and the Internet of Things as representatives of the industrial production field [1]. Intelligent manufacturing [2] is a major player in promoting future industrial development. Increasing efforts to drive the development of the intelligent manufacturing industry is not only a key measure to realize a strong manufacturing country but also a core element in promoting the national manufacturing industry to comply with global trends, upgrade its technology, and achieve leapfrogging.

**Figure 1:**
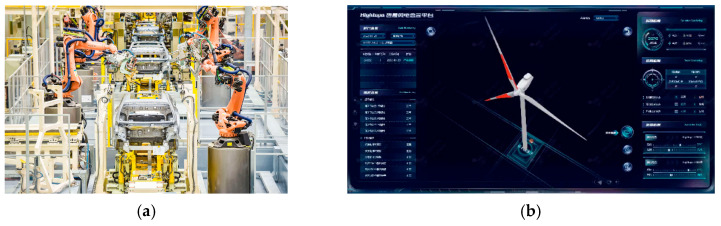
(**a**) GAC new energy intelligent eco-factory. (**b**) Wind turbine operation status monitoring.

In recent years, countries have made efforts to promote the in-depth integration of digital capabilities with production and manufacturing capabilities, comprehensive coherence between the digital economy and real industries, deep synergy between the informatization process and the development of industrialization, and the close integration of artificial intelligence technologies with the manufacturing sector [3]. After years of cultivation, the world’s intelligent manufacturing has made great progress, and the intelligent manufacturing industry is gaining significant results in multiple fields and scenarios. Figure 1a shows a new energy automobile manufacturing workshop, with the help of intelligent equipment, robotics, Internet of Things, and other technologies, to achieve intelligent upgrading of the production line, improve the efficiency of the production capacity, reduce production expenditures, and promote the refinement of production [4]. As shown in Figure 1b, relying on an intelligent inspection system, the operation status of large wind turbines can be monitored through the cloud even from thousands of miles away [5].

The concept of digital twins was first introduced in 2002, when Dr. Michael Grieves, an American doctor, proposed using computers to create a model that is identical to a real-life object. After that, it was not until 2010 that a practical application was developed [6]. At that time, performing space missions was complex and extremely risky, and the cost of physical testing and validation before the mission could take place was extremely high. In order to ensure the successful completion of space missions while reducing testing costs, NASA needed a technology that could accurately simulate the flight status of space vehicles. At this time, NASA’s Prof. John Vickers successfully constructed digital twin models of space capsules and vehicles using digital twin technology, and conducted a series of tests and simulation experiments through these models [7]. After 2015, with the rapid development of artificial intelligence algorithms and virtual reality technology, digital twin technology began to be applied in the industrial field. In 2017, digital twin technology was listed as one of the top ten strategic technology trends by Goldner Technology Consulting, while companies such as Siemens, Boeing, and General Motors all launched relevant projects to develop digital twin models. After 2017, digital twin technology moved to further industrial applications, fully expanding from aerospace and industrial manufacturing [8] to various industrial fields [9] such as transportation [10] and healthcare [11]. In the manufacturing industry, the loss of mechanical equipment can be predicted by digital twins, and maintenance plans can be developed [12]. In the medical field, digital twins can simulate the function of human organs, help doctors accurately diagnose diseases, and promote the development of personalized medicine. In the transportation industry, digital twins can simulate the traffic system of the flow of people and vehicles, providing a reference basis for the development of urban transportation planning.

As a cutting-edge technology with unique application value and potential, digital twin technology has already shown remarkable performance in many fields [13]. In order to reduce production costs and ensure the safe operation of welding robotic arms, data-driven digital twin technology has been introduced into the production process of welding robotic arms [14]. Through high-precision 3D models, highly reproducible working environments, and multi-module human–computer interaction interfaces, with the help of data transmission technology and scripts, a welding robot arm digital twin system has been designed and developed. In the welding robot arm digital twin system, the robot arm’s movement route can be verified through simulation. Before putting the robot arm into actual production, the simulation results are analyzed to predict the problems and errors that may occur. This simulation process not only improves the accuracy of trajectory planning but also effectively prevents productivity and product quality problems caused by improper planning. In addition, the data-driven digital twin of the welding robot arm can update the parameters of the robot arm production process in real time, through real-time monitoring of the welding process to detect the dynamic changes that occur in production in a timely manner. The digital twin system can respond quickly to abnormal situations, intervene in the production process, analyze the cause of the problem, and adjust the trajectory to ensure safe production. For example, changes in the temperature of the welding torch, current, voltage, etc., beyond the normal range will lead to poor welding quality, and more serious safety accidents can occur.

## 2. Program Design for Building a Digital Twin System for Welding Robot Arms

**Figure 2:**
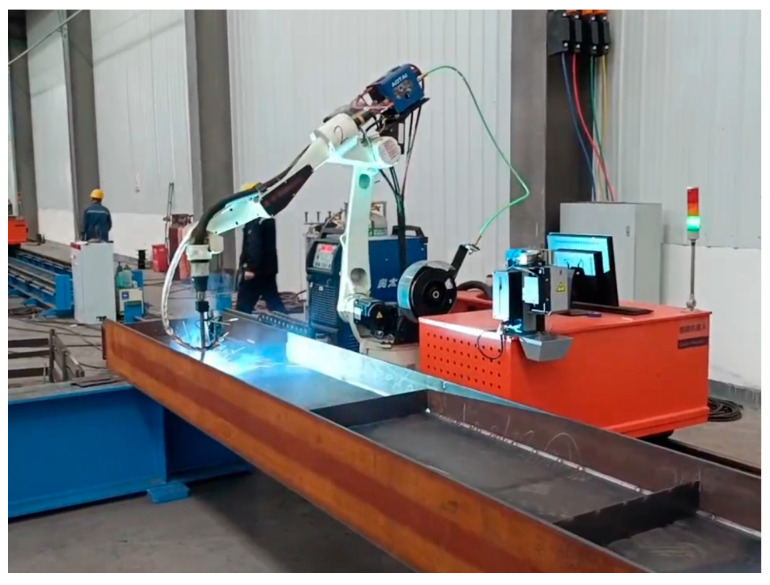
Welding robotic arm table.

The configuration of the welding workstation equipment is shown in Figure 2. The workstation integrates a welding robotic arm, ground track drive motor, welding power supply, welding wire spool, human–machine interface, robotic arm control system, and other components, achieving a high degree of integration. This integrated approach, which combines all equipment on a single platform, reduces the need for external cables and piping connections, thereby alleviating the labor intensity during installation and commissioning. This effectively enhances the overall operational efficiency and reliability of the equipment.

The mobile ground track system of the welding workstation consists of components such as the ground track base frame, precision-ground steel rails, and drive racks. Driven by an external shaft motor located at the bottom of the welding workbench, the workbench can move horizontally along the ground track via rollers. The six-degree-of-freedom robotic arm itself has sufficient flexibility in movement. When combined with a movable welding platform, the welding robotic arm can effectively expand its reachable range. When integrated with an intelligent welding system to plan welding paths, it can adapt to welding components of various sizes, particularly when welding long-span structures. The entire welding robotic arm workstation has a high degree of integration, is simple to operate in practice, and has largely achieved automated welding, with welding quality meeting production requirements.

The welding arm digital twin feeds production data from the actual welding environment into the virtual end model for real-time monitoring, alerts, and optimization of the welding arm’s path trajectory. In addition, the user can modify parameters in the simulation module of the digital twin to reflect changes and challenges that may occur in actual production. This makes the digital twin not just a static simulation tool, but a tool that can dynamically adjust and optimize the production process.

**Figure 3:**
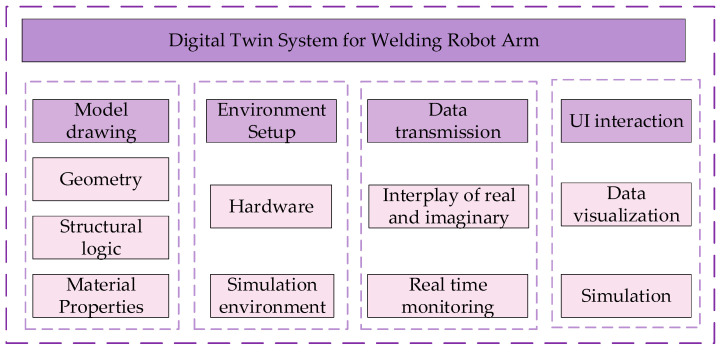
Digital twin system architecture for welding robotic arm.

The digital twin system architecture for the welding robot arm is shown in Figure 3.

The construction of the welding robot arm digital twin system is divided into the following four parts: model construction, environment building, data transmission, and UI interaction.

(1) Model drawing: Before drawing the model, we must first ensure that we obtain accurate model data. When restoring the real model, not only should the geometry of the physical model be restored, but its structural logic and material properties should also be considered. The accuracy of the model is pivotal to the subsequent simulation and prediction of the digital twin of the welding robot arm.

(2) Environment construction: The environment construction includes two aspects: One is that the computer environment construction, the model rendering, data transmission, and UI interaction functions of the digital twin system have very high requirements for hardware facilities, so we need to establish a computer environment that can support the normal operation of the digital twin system. The other aspect is the simulation environment; in order to make the virtual end model able to completely simulate the real situation, we also need to restore the real production environment.

(3) Data transmission: Data transmission is the key to realizing the operation of the welding robot arm digital twin system. The real-time data will be transferred from the physical entity to the virtual end model, and then the prediction and optimization strategies generated by the model will be fed back to the physical entity.

(4) UI interaction: The UI interaction interface is not only the bridge between the user and the virtual end model but also the core of realizing data-driven physical entities and outputting virtual end commands.

### 2.1. Digital Twin Modeling

The digital twin model can reflect the physical system, process the real world in real time, and perform simulation, modeling, and prediction through real-time data updates and feedback. Therefore, the accuracy of the virtual mapping of the digital twin system depends on the accuracy of the data transmission on the one hand, and on the other hand on whether the model construction meets the requirements.

**Figure 4:**
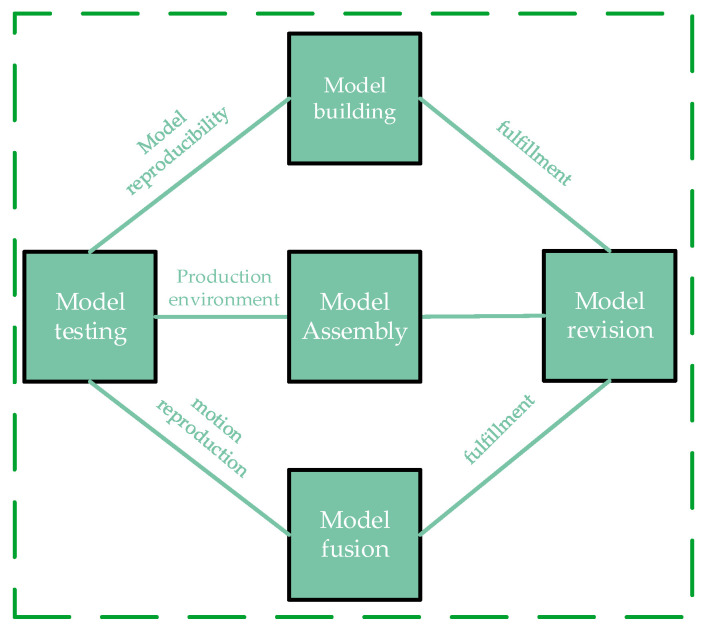
Digital twin model construction scheme.

Digital twin model construction consists of the following five parts: model construction, model assembly, model fusion, model testing, and model correction (Figure 4).

Model construction requires a multidimensional and multifaceted approach and a high degree of consistency with the physical entity. Subsequently, the model is assembled, the model hierarchy is constructed, the parent–child relationship between parts is clarified, and appropriate constraints are added to facilitate the actual production process. Model integration refers to the relatively complex production environment; the model construction needs to be integrated into the models of different fields and disciplines. For example, in this paper, the welding robot arm model construction needs to take into account the mechanical system, control system, and electrical system. After completing the above steps, the model needs to be tested in all aspects, and when the test results do not meet the actual needs, the model should be corrected accordingly, and then retested until it meets all the requirements.

### 2.2. Digital Twin Modeling of Welding Robot Arm

When constructing the digital twin model, special attention is paid to the one-to-one restoration of the dimensional structure and motion characteristics of the robot. The dimensional structure of the robot arm should be restored with full consideration of the relative position of each component. The welding robot arm is manufactured by Kawasaki, and its relevant data can be obtained directly. The data for the support of the weldment, the ground rail, and the operating table should be measured accurately, with particular attention paid to the distance between the ground rail and the weldment being welded. A slight deviation from this distance changes the working range of the welding robot arm.

When restoring the motion characteristics of the robotic arm, two aspects need to be considered. One is the action modeling, that is, considering the range of motion and kinematic constraints of the robotic arm joints, and limiting the range of motion of each joint of the robotic arm in the virtual model to be consistent with Table 1. On the other hand, the control relationship between the joints of the robotic arm is considered. Each joint is not an independently existing individual, so a parent–child hierarchical logical relationship is added when restoring the robotic arm model. By constructing the digital twin model with high precision, the motion characteristics of the robot can be reproduced realistically. This provides a reliable simulation tool for robot control, path planning, and behavior prediction based on digital twin technology.

The three most commonly used modeling methods for constructing digital twin models are as follows: 3D modeling software (SolidWorks 2022, Blender-3.6.2, etc.), industrial-grade instrumentation modeling (laser scanners, 3D scanners, etc.), and modeling based on photos or on-site videos (combining image processing and computer vision technologies). During the initial modeling phase, we attempted to use a 3D scanner for modeling. However, this approach revealed several issues: 3D scanners can only scan the overall structure of the equipment and cannot reproduce detailed features. Scanning individual parts is labor-intensive and disrupts normal production operations. SolidWorks 2022 software, widely used in mechanical design, engineering modeling, and manufacturing, can directly utilize CAD data provided by manufacturers to enable precise geometric modeling. Ultimately, to meet the requirements of high-precision data and achieve a highly reproducible real-world model that aligns with actual production processes, we utilized SolidWorks 2022 3D modeling software to construct the 3D model of the welding robotic arm.

After completing the 3D model of the welding robotic arm, Unity3D 2022.3.0f1c1 was used as the platform for building a digital twin. Unity 3D 2022.3.0f1c1 offers fast 3D modeling and visualization capabilities, supports multi-format model compatibility and lightweight rendering, and enables dynamic simulation through its physics engine. It excels at data interaction and communication integration, facilitating bidirectional data transmission between virtual end points and physical devices via multi-protocol communication interfaces. It can also integrate with tools such as MATLAB R2022a, SQL Server Management Studio 19, and others. It offers rapid low-code development and interactive design advantages, leveraging a modular UI development toolchain and immersive scene navigation features, making it suitable for developing virtual training systems. It boasts industrial-grade scalability and ecosystem support, with a wealth of third-party plugins and cross-platform deployment capabilities. It also has a certain degree of compatibility with industrial standards, ensuring time synchronization and precise control, and can connect to industrial devices such as PLCs via protocol plugins. Although it has limitations in terms of industrial-grade real-time performance, it offers high development efficiency, high simulation accuracy, and low-cost barriers, making it the optimal platform for rapid validation, low-cost trial-and-error testing, and high-interactivity scenarios in the industrial digital twin field. Additionally, by leveraging Unity’s low-code ecosystem, it enables the widespread adoption of DT technology among small and medium-sized enterprises, as complex custom development systems are economically unfeasible for such businesses.

**Figure 5:**
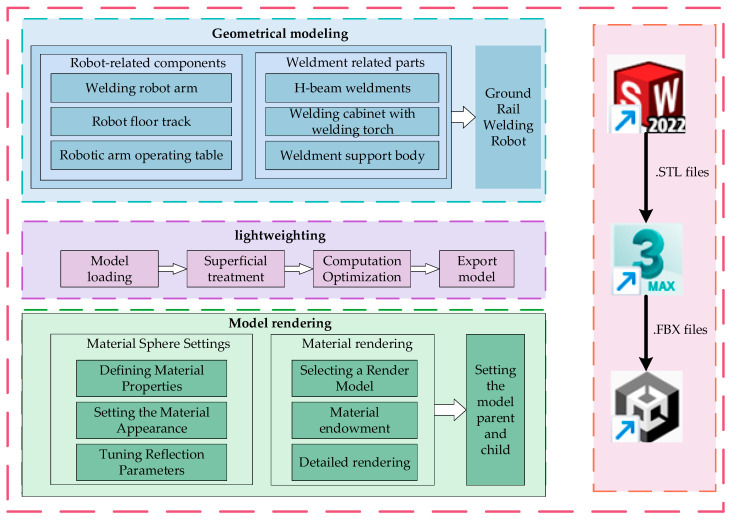
Digital twin model construction process.

The process of constructing the digital twin system model for the welding robotic arm is illustrated in Figure 5.

As shown in Figure 5, the construction of the welding robot arm digital twin system involves the transfer of models between the three software platforms. The model created in SolidWorks is saved as an “. STL” file, and the “. STL” file is converted to an “. FBX” file in 3dsMax 2021 and Unity 3D 2022.3.0f1c1.

**Figure 6:**
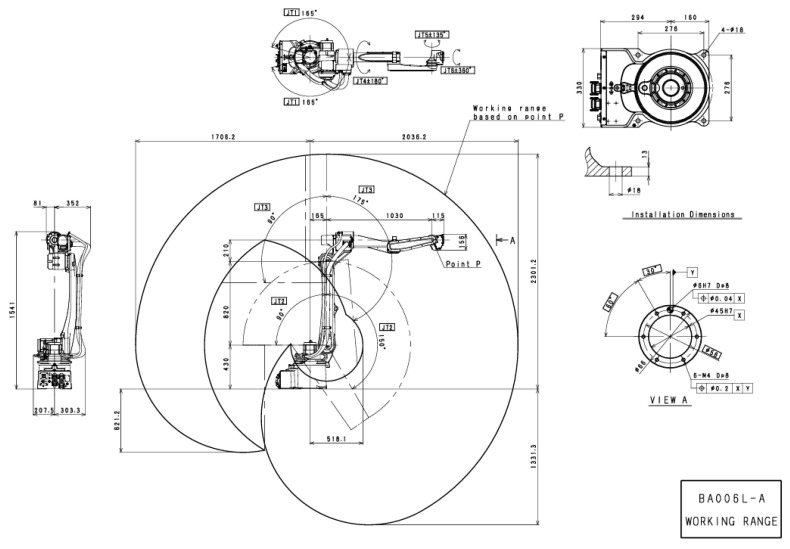
**CAD** data diagram of BA006L robotic arm.

**Figure 7:**
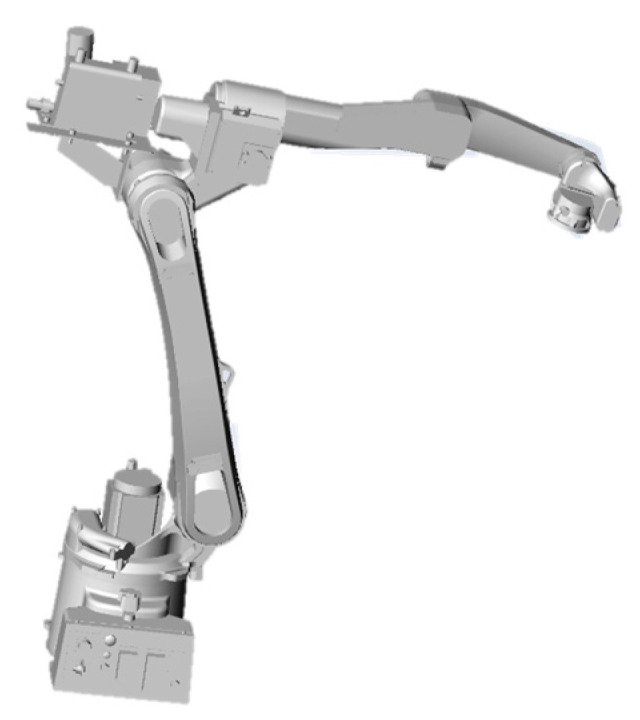
Geometric model of welding robot arm.

When using SolidWorks software to build a welding robotic arm model, we should fully consider the mechanical structure of the robotic arm and the assembly relationships between its components. We assemble the various components of the welding robotic arm, such as the arm, joints, and base, to form a complete robotic arm structure. After modeling is complete, we use SolidWorks’ motion simulation functionality to verify the robotic arm’s motion performance. The robotic arm used in actual production is the Kawasaki BA006L welding robot, whose CAD drawings are shown in Figure 6. The three-dimensional model created based on the CAD drawings is shown in Figure 7.

**Figure 8:**
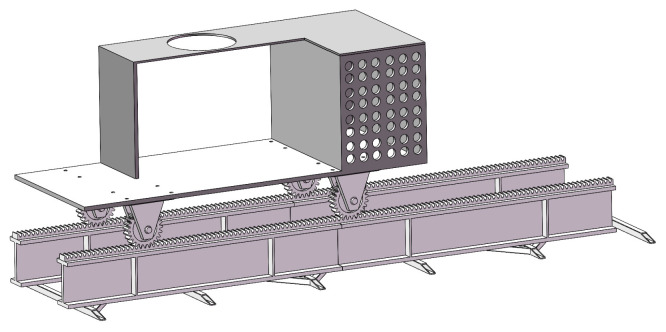
Ground rail assembly.

After completing the drawing of the robot arm model, the next step is to continue to draw the ground rail, gears, rack and pinion, operating table, and other related models, and they will be assembled together in accordance with the actual production relationship to ensure that they are highly compatible with the actual production, and ultimately form an overall assembly, as shown in Figure 8.

**Figure 9:**
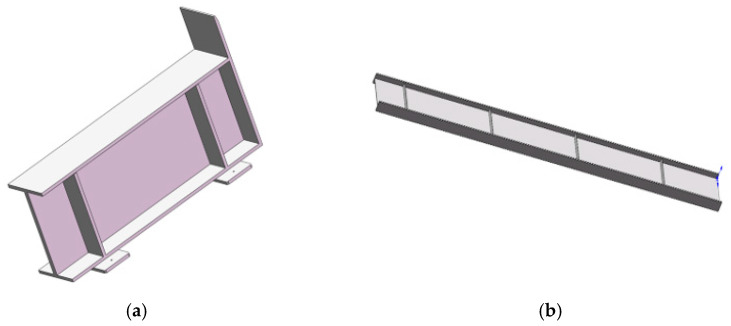
Three-dimensional models of welding support and weldment. (**a**) Weldment support. (**b**) Weldment.

The next step is the modeling of the weldment as well as the support of the weldment, and the 3D model is shown in Figure 9.

### 2.3. Model Lightweight Processing

The actual production size of welding workstations is too large, and because of the complex structure of the robot arm model, there are too many models on the triangular surface, occupying too much memory. In the whole process of digital twin system development, the above situation will require too much performance, which will lead to the system reading the model incompletely and lag in the operation, so a twin model is required for lightweight processing. The processed model should retain geometric information such as size, assembly relationship, and material properties, consistent with the physical entity, in addition to ensuring that the details of the part are not lost or deformed, to ensure that the model’s inside and outside have no deformations, cavities, or other problems.

(1) Edge Shrinkage

**Figure 10:**
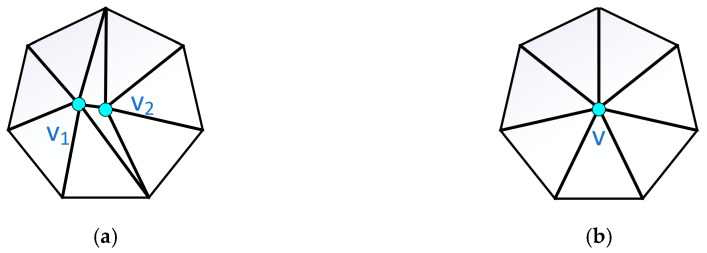
Results before and after edge shrinkage. (**a**) Pre-systolic. (**b**) Post-systolic.

Three-dimensional models are essentially composed of triangular faces, and the most direct and effective way to lighten the model is to reduce the number of triangular faces of the model. The end result of edge contraction is to synthesize two vertices into one vertex, as shown in Figure 10.

According to Figure 10, points V1 and V2 are the two internal vertices of the model. The smallest edge is selected as the shrinkage cost minimization factor for vertex merging—merging V1 and V2 generates a new vertex V. As an internal vertex, V connects to the remaining vertices to form new triangular faces, thereby reducing the number of triangular faces. After multiple iterations of this process, the model is finally lightweighted by systematically minimizing the triangular face count through successive vertex merges.

(2) Minimizing Deformation Degree

In the process of lightweighting, the minimization of the degree of deformation is used as a vertex contraction condition, and the “degree of deformation” of vertex V is described by the quadratic error function. In the triangular mesh in three-dimensional space, when the position of a vertex V changes, the distance from the vertex V to the face must change. The square of the distance from the point to the face is the degree of deformation of the point, that is, the quadratic error function, assuming that each vertex used to measure the face planes (v) is not unique. The plane is obtained through the formula $a_{x}+b_{y}+{\;c}_{z}+d=0$, and the formula satisfies $a^{2}+{\;b}^{2}+{\;c}^{2}=1$. Suppose that the triangulation plane $p={\left[a,\;b,\;c,\;d\right]}^{T}$, the vertex $v={\left[v_{x},\;v_{y},\;v_{z}\right]}^{T}$, and the vertex at the triangulation plane by the distance D squared is

(1) $$D^{2}={\left(p^{T}v\right)}^{2}=v^{T}(pp^{T})v$$

with planes (v) representing the ensemble of all metric faces.

(2) $$Q\left(v\right)=\sum_{p\text{∈}\mathrm{planes}(v)}pp^{T}$$

From Equations (1) and (2), the quadratic error function is given by

(3) $$\text{∆}\left(v\right)={\;v}^{T}\mathrm{Qv}$$

thus minimizing the degree of deformation:

(4) $$\mathrm{minimize}\sum_{v}V^{T}\mathrm{QV}$$

To minimize the deformation, we first calculate the initial Q of each vertex, which satisfies $v^{T}\mathrm{Qv}=0$. We keep all the pairs of points that satisfy the condition, calculate the above pairs of points according to the quadratic error function, and choose the minimum value of $\text{∆}\left(v\right)$. The mesh is then updated with all the vertex positions. The process is repeated for the updated model and stopped when the model meets the target number of points.

**Figure 11:**
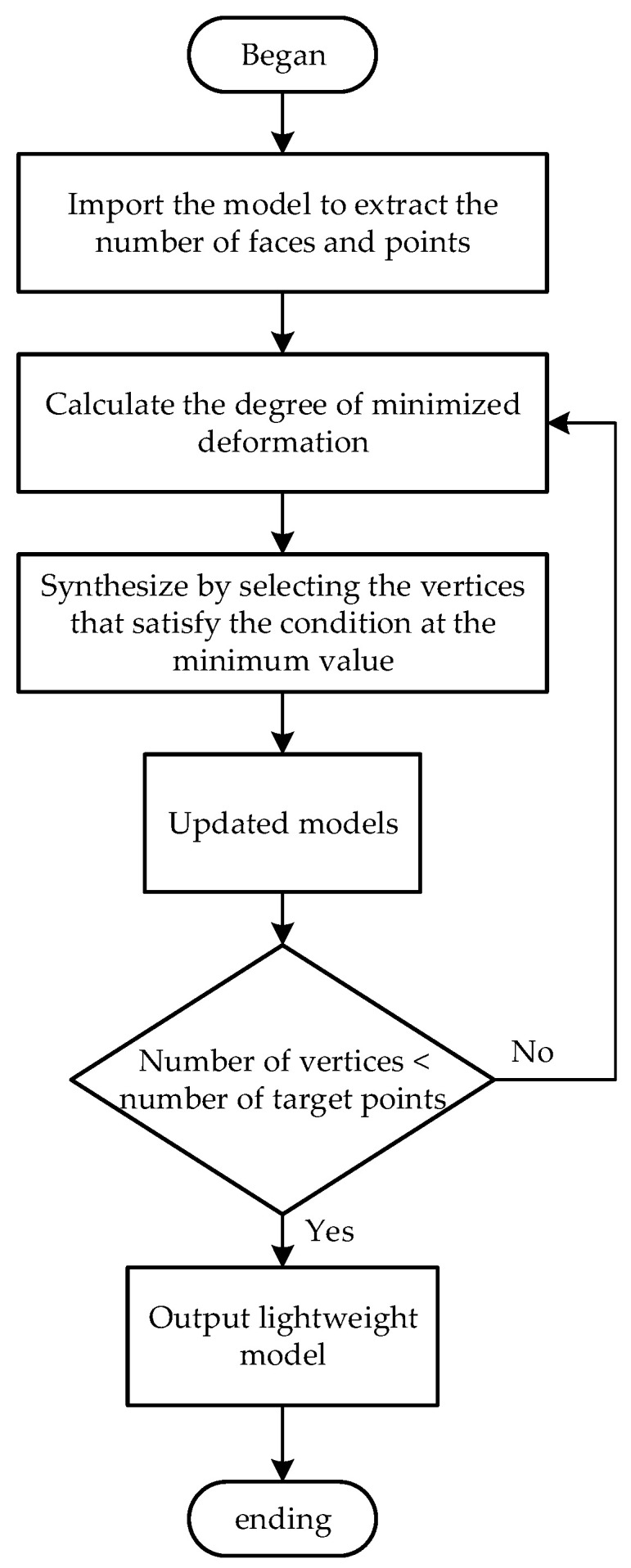
Flowchart of the model’s lightweight processing algorithm.

The algorithm flow is constructed based on the quadratic error function according to minimizing the degree of deformation as the cost of edge contraction, see Figure 11.

Step 1: Import the 3D model, and read the model to extract the triangular faces and vertices.

Step 2: Calculate the normal vector of the vertices, and then calculate the minimized deformation degree function according to the normal vector.

Step 3: Select the vertices that satisfy the condition when “∆(v)” is minimized for synthesis.

Step 4: Update the model. If the number of vertices in the model reaches the target number, proceed to step 5; otherwise, repeat step 2.

Step 5: Output the model.

**Figure 12:**
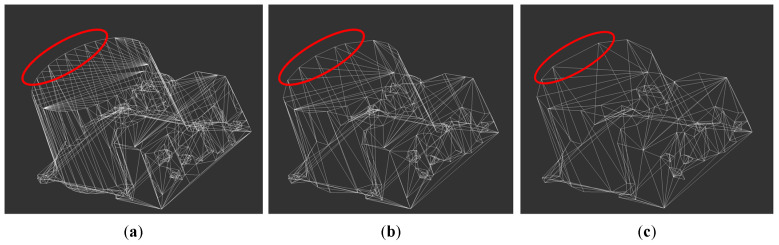
Lightweighting of the robotic arm base. (**a**) Vertex not contracted; (**b**) 50% shrinkage in the number of vertices; (**c**) 70% shrinkage in the number of vertices.

**Figure 13:**
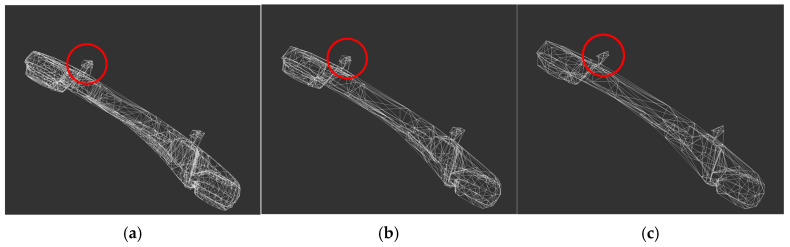
Lightweight treatment of robotic arm joint q1. (**a**) Vertex not contracted; (**b**) 50% shrinkage in the number of vertices; (**c**) 70% shrinkage in the number of vertices.

Finally, the models of the robotic arm base and joint q1 are processed by the lightweighting algorithm, and the percentage of vertex shrinkage is set to 50% and 70%, respectively, to compare the models before and after processing, as shown in Figure 12 and Figure 13.

After comparing the effect before and after the model lightweighting treatment, it is found that when the number of vertices is contracted by 70%, the circular edge diameter becomes irregular, so the number of vertices is contracted by 50% when the model lightweighting treatment is carried out, which can reduce the number of triangular faces of the model to optimize the system, and at the same time ensure that the model is not distorted. All models are lightweighted, and the results are shown in Table 2.

### 2.4. Model Rendering

**Figure 14:**
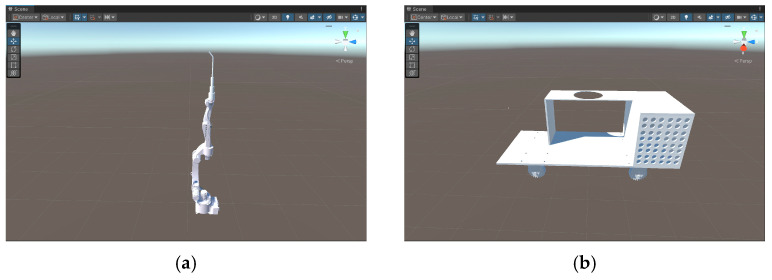

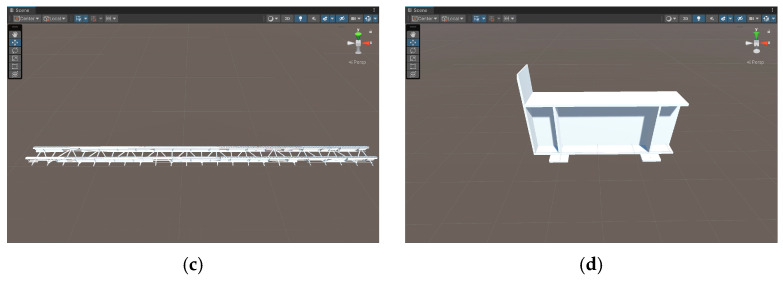
Initial state of the model in Unity 3D. (**a**) Welding robot arm. (**b**) Control panel. (**c**) Underground railway track. (**d**) Weldment support.

The model is imported into Unity 3D before it is rendered. The process of format conversion has been described in detail in Figure 3. In this process, the default world coordinate system orientation is different between 3dsMax and Unity 3D, so the coordinate system must be converted accordingly in 3dsMax before importing. The original parts imported into Unity 3D are shown in Figure 14.

**Figure 15:**
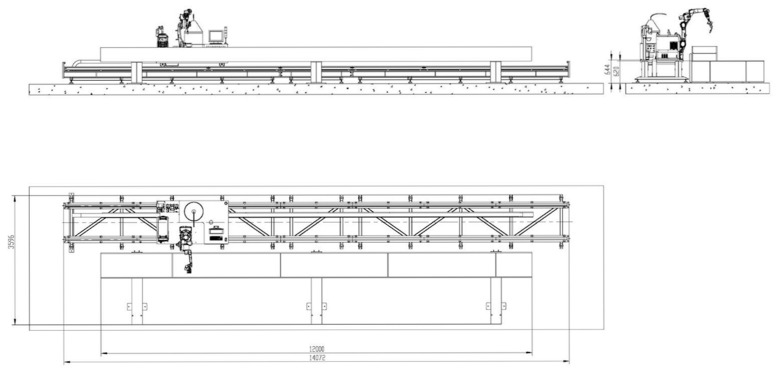
Equipment layout diagram.

**Figure 16:**
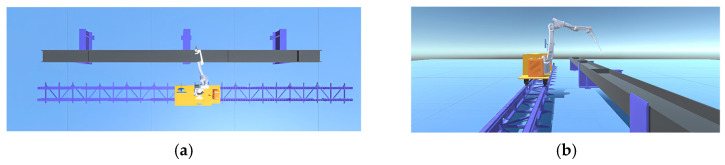
Model effect in Unity 3D. (**a**) Top view of the model. (**b**) Left view of the model.

Rendering models involves not only considering the model’s visual attributes but also its material properties. Models are rendered using the material component in Unity 3D. Each material component contains properties that describe the object’s appearance and material attributes, such as commonly used metallic materials in machinery, concrete materials in construction, and glass materials. Developers can configure appropriate appearance and material attributes by applying different materials to game objects. Additionally, developers can create their own materials using shader scripts to meet specific requirements. After creating the desired material, the model is rendered, and the scene is constructed based on the actual production environment (Figure 2) and device layout diagram (Figure 15), with the final rendering of the actual effect in the Unity 3D engine as shown in Figure 16.

### 2.5. Parent–Child Hierarchy Logic Setting

Parent–child hierarchy logic refers to the movement relationship between two or more game objects. A game object can be set up as a parent by more than one child object. Once a parent–child relationship is established between them, the parent object is no longer independent. When changing various attributes of the parent object, such as position, rotation, and scaling, the child object will inherit the attribute commands of the parent object and make the corresponding changes. The setup of parent–child hierarchical logic not only simplifies the management and operation of the game objects, but also reflects the kinematic relationship between the objects, especially in the welding robot arm, which is more obvious.

**Figure 17:**
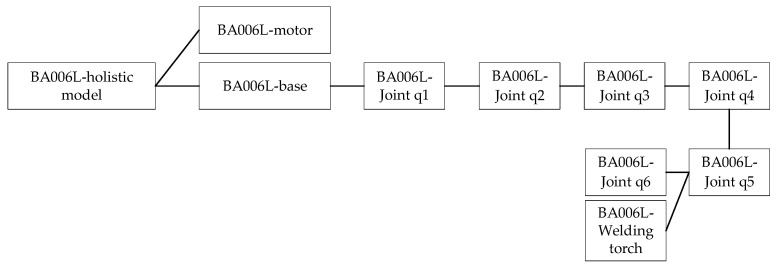
Robotic arm paternity.

The parent–child relationship in the scenes that appear in this paper is mainly considered a logical correlation. After importing the model into the scene, the interactions between the parts of the robotic arm model need to be considered in order to conform to real physical laws. There is a connection between the parts of the robotic arm entity; for example, when joint q1 rotates around the Z-axis, the rest of the joints have to rotate with joint q1 while ensuring that the relative position remains unchanged. The parent–child relationship of the mechanical arm is set up as shown in Figure 17.

Among them, the whole BA006L model does not contain any model parts, and only serves as the parent of all robotic arm parts, which is convenient for the moving, rotating, scaling, etc., of the whole model. The levels in the figure show the parent–child relationships; the middle level, which is the child relationship of the top level, also represents the parent relationship of the bottom level.

## 3. Digital Twin Model Testing

After completing the model construction, the robotic arm needs to be tested from multiple angles to verify that it meets all the requirements when performing the specified production actions and to ensure that it can complete the task successfully. In order to achieve better production results in Unity 3D, collision detection and particle effects simulating the actual welding process were added.

### 3.1. Collision Detection

In Unity 3D, collision detection is implemented through the physics engine. In Unity 3D, each game object can be attached to one or more colliders, which are generally used to approximate a complex physical object as a square box used to define the collision shape of the game object. The better the collision area fits the shape of the object, the higher the collision detection accuracy. In this system, the model involved does not have a specific shape, so we choose Axis-Aligned Bounding Box (AABB), which fits the model better. The bounding box is hexahedral and parallel to the coordinate axis, and the length of any of its edges can be adjusted. It is expressed by the following formula:

(5) $$R=\left\{\left(x,y,z\right)\left|X_{min}\leq X\leq X_{max},\right.Y_{min}\leq Y\leq Y_{max},Z_{min}\leq Z\leq Z_{max}\right\}$$

$X_{min}{,\;Y}_{min},\;Z_{min}$ denote the minimum value of X, Y, and Z corresponding to the enclosing box, respectively. $X_{max},\;Y_{max},\;Z_{max}$ denote the maximum value of X, Y, and Z corresponding to the enclosing box, respectively.

**Figure 18:**
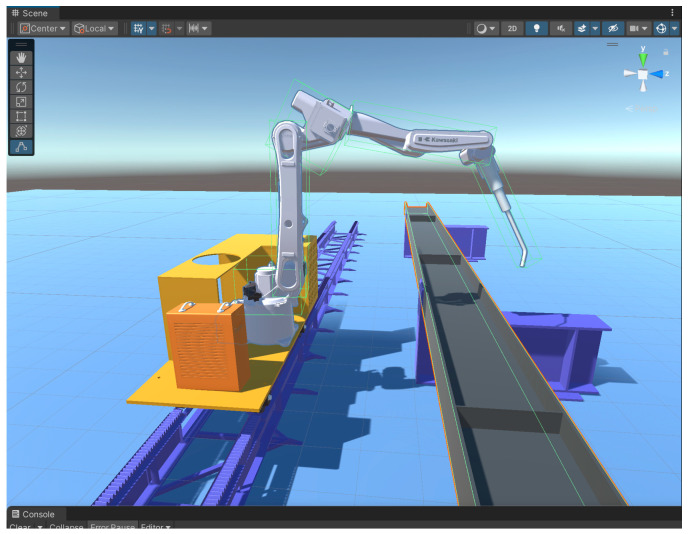
Collision body effect of robotic arm and weldment.

At the same time, objects need to be labeled as rigid bodies in order to participate in the physical simulation. By analyzing the actual production process, only the collision between the robotic arm and the weldment is considered here. The collision body is added to the robot arm and the weldment separately, as shown in Figure 18, and the green border around the object is the collision detection range. We write the corresponding C# script to call each function to realize the collision detection and collision tips.

**Figure 19:**
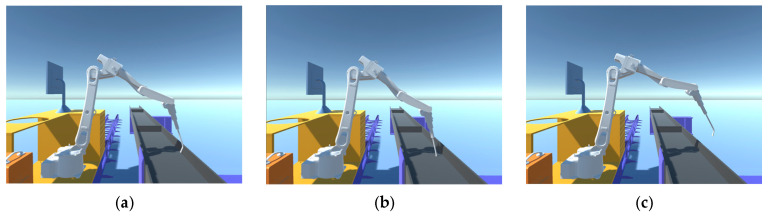
Welding robot arm collision detection. (**a**) Entering the collision zone. (**b**) Collision zone. (**c**) Leaving the collision zone.

The script will utilize the physics engine to periodically perform discrete collision detection between the robotic arm and the weldment; each collider will periodically emit rays in all directions. The motion trajectory of the welding robot arm is computed by the physics engine within a defined time node, and the motion trajectory is also detected for collisions. When the collision body on the robotic arm is in contact with the collision body on the weldment, a collision occurs, and the console interface prints a collision prompt, as shown in Figure 19. When the robotic arm is 1cm from the weldment, the console interface prompts the user to enter the collision area. When the robotic arm and the weldment intersect in the collision area, the console displays that a collision is occurring. When there is no contact between the robot arm and the weldment, the console interface prompts the robot to leave the collision area.

In addition, Unity 3D can utilize the collision detection callback function to set off the next commands, such as playing audio or video, triggering particle effects, or triggering specific actions.

### 3.2. Welding Particle Effects

In order to make the virtual end of the digital twin closer to the actual production, the particle system in Unity 3D is used to simulate the effect of sparks and flames. The effect of sparks spattering during welding is simulated by adjusting the color, shape, light, range, and other properties of the particle system components. The setting parameters are shown in Table 3 below.

**Figure 20:**
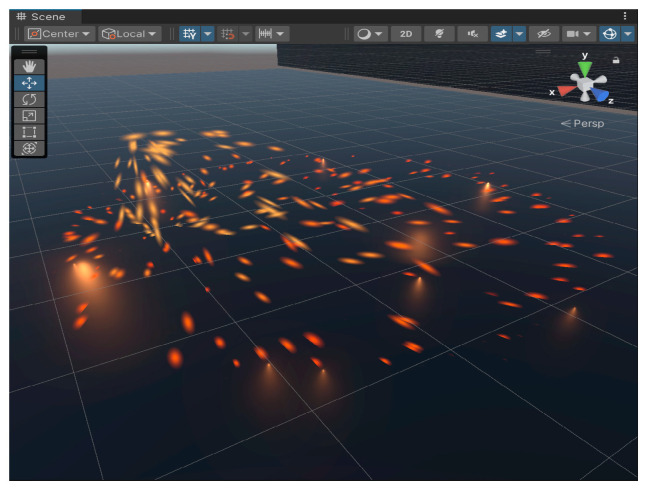
Welding particle effect.

**Figure 21:**
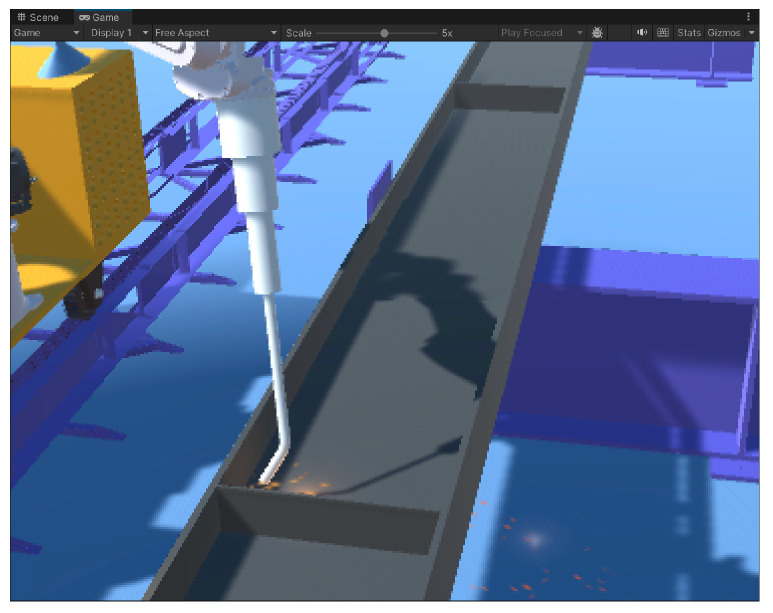
Sparks triggered by collision.

Through the above parameter settings, the welding particle effect with spark and flame effects is created in Unity 3D as shown in Figure 20. The welding particle effect is added to the welding robot arm model, using the C# language to write the collision event callback function that is triggered when a collision occurs, triggering the particle effect. A collision box is added to the end of the welding gun; when the collision box is in contact with the weldment, the particle effect is triggered. The collision trigger spark effect is shown in Figure 21.

### 3.3. Robotic Arm Positioning Test

In the entire welding production model, in addition to the lateral motion involved in the ground rail, the robotic arm, as a mechanical production equipment with complex motion characteristics, needs to ensure that the virtual end model can successfully complete the specified movements before connecting with the physical end. The simulation module in the digital twin system uses the keyboard to control the rotation or translation of the joints of the virtual arm through the writing of scripts to complete the specified movements. The joints of the welding robot arm have different axes of motion; for example, the arm joint q2 rotates around the X-axis at the origin of the virtual end. Before writing the corresponding script, it is necessary to clearly assign the axes of motion to each joint of the robotic arm and the mode of motion, so as to prevent the confusion caused by the inability to realize the correct movement of the robotic arm. In addition, in the process of controlling the movement of the robot arm, both local and global coordinate systems are involved. It should be noted that the rotational movement of the robot arm is carried out around the local coordinate system (i.e., the coordinate origin of the component), while the transverse movement of the console takes the world coordinate system as a reference.

The reference coordinates of each moving object and the assignment of the axes of motion are shown in Table 4 below.

**Figure 22:**
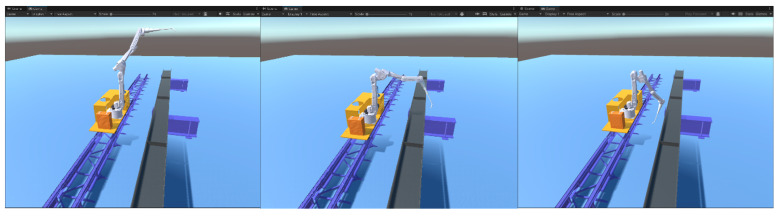
Robotic arm position test.

The model was tested after the above preparations were completed, and the robotic arm position test is shown in Figure 22. The model did not deform, and the end-effector of the robotic arm could reach the specified position.

## 4. Data Transmission of Digital Twin System for Welding Robotic Arm

The modeling of the digital twin system of the welding robotic arm, as well as the environment construction, was completed in Section 2 and Section 3. Next, the last two parts of the welding robot arm will be completed: data transfer and UI interaction. Data transfer refers to the transfer of welding-related data from the physical side to the virtual side, which further enables the digital twin to monitor the actual production in real time, while UI interaction is the design of the UI to visualize the above data. In addition, the UI integrates the various functional modules of the digital twin together, so that users can directly call the relevant commands. This chapter gives a detailed description of the research on realizing the data-driven digital twin UI for welding robotic arms.

### 4.1. Design of Data Transmission Scheme for Digital Twin System of Welding Robotic Arm

**Figure 23:**
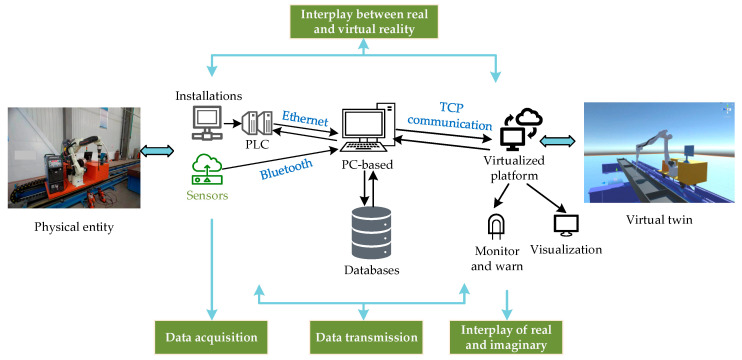
Interaction process framework of digital twin system for welding robotic arm.

The data connection between the welding robotic arm digital twin system and the physical entity includes data acquisition, data transmission, virtual reflection of the real robotic arm, and real control of the virtual model. The specific process is shown in Figure 23.

(1) Data Acquisition: Data acquisition refers to the organization and collection of the data of the welding robot arm workstation in the actual production process. The real-time acquisition of data is realized through a variety of data acquisition terminals, mainly based on sensors. According to the different types of actual on-site equipment, the appropriate communication protocol between the equipment is selected, such as Modbus, TCP, Ethernet, Bluetooth, or Wi-Fi.

(2) Data transmission: Data transmission is a key link in the realization of digital twins, and it is the basis that enables the virtual end to reflect the physical end. The data transmission part of the welding robot arm digital twin system involves two transmission processes. One is the data transmission between the digital twin system and the physical entity, and the other is the data transmission between the algorithm software on the PC side and the digital twin system.

(3) Virtual image of real robotic arm: The data collected by the field equipment is transferred to the digital twin through various communication methods, and then the actions of each link of the welding robotic arm workstation are accomplished according to the data types, including the traveling and stopping of the ground rail, the rotation of each joint of the robotic arm, and so on.

(4) Virtual control of the real robotic arm: The ability of the welding robotic arm digital twin system to control the physical entity is key for realizing its remote-control operations. The data transmission channel will regulate the commands or information output to the physical entity, so as to control the field equipment to complete the specified command. The realization of virtual control meets the requirements of saving resources, and at the same time, prevents casualties due to operational errors, to ensure that the production site is safe and reliable.

Data transmission is the key to enabling the welding robotic arm digital twin system to connect to the physical entity. The data transmission between the virtual end and the physical entity needs to meet the following conditions:

(1) Real-Time Transmission: Real-time transmission can ensure that the system accepts the data from the physical end in real time, to ensure that the welding robotic arm’s physical entity sends the virtual twin real-time feedback. Real-time feedback between the virtual and real robotic arm can enable the digital twin system to quickly reflect the changes in the operating status of the equipment, and adjust the production strategy and operating parameters in a timely manner to optimize production efficiency and quality.

(2) Accuracy: The accuracy of data transmission directly affects the reliability of the welding robot arm digital twin system. This requires the actual collection of data to ensure that the data is accurate and complete, while ensuring that the data is not lost in the transmission process and is not affected by noise.

### 4.2. Welding Robot Arm Data Acquisition and Transmission

**Figure 24:**
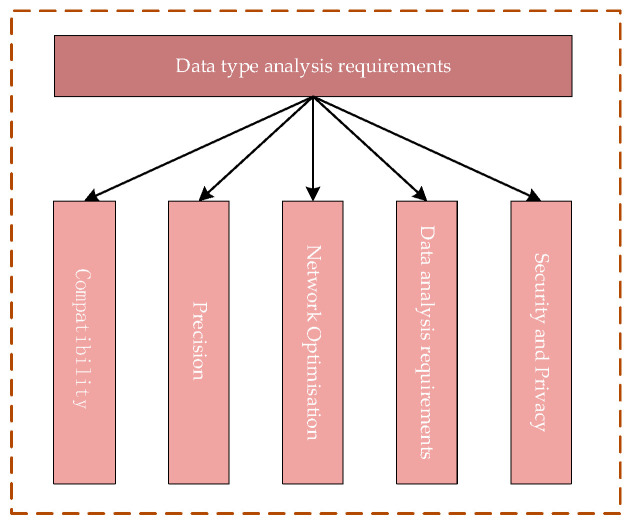
Data type analysis requirements.

The entire welding process is a collaborative effort of mechanical and electrical systems, involving multiple data types. The process of data acquisition and transmission involves various layers, such as a physical layer, virtual layer, and data layer. Before collecting data for data type analysis, the following five points should be considered (Figure 24) [15].

(1) Data compatibility: Data compatibility refers to different communication methods in the transmission of data. The process of data formatting may be different, so the collection of data should be fully considered before the data is collected to ensure that it is compatible with the current system. Data format mismatch should be avoided to prevent data loss or failure to parse the situation.

(2) Data accuracy: First, the accuracy of each data type should be determined before data transmission. The required accuracy, compression, or streamlining of the data should be within the allowable range to avoid the problems of slow operation or system overload.

(3) Encrypted data security: Some of the data in the digital twin system may involve corporate secrets or private personal data. Analyze whether there is any private data before data transmission, use a separate data transmission channel for confidential data, and at the same time, through encryption of data, restrict access authorization. This is to ensure that the data is transmitted securely and privately.

(4) Data processing and analysis needs: In the digital twin system, different data types represent different information, and before data collection, it is necessary to understand the use and needs of each data type, so as to involve appropriate data processing processes. For example, depending on the frequency of data updates, some data types need to be analyzed and processed in real time, while certain types only need to be updated periodically or stored for backup.

(5) Network and bandwidth optimization: When transferring large amounts of data through the cloud or remote servers, network bandwidth requirements are extremely high. The frequency and magnitude of data transmission should be determined when categorizing the data, and data of the same frequency and magnitude can be transmitted together to reduce costs and improve efficiency.

**Figure 25:**
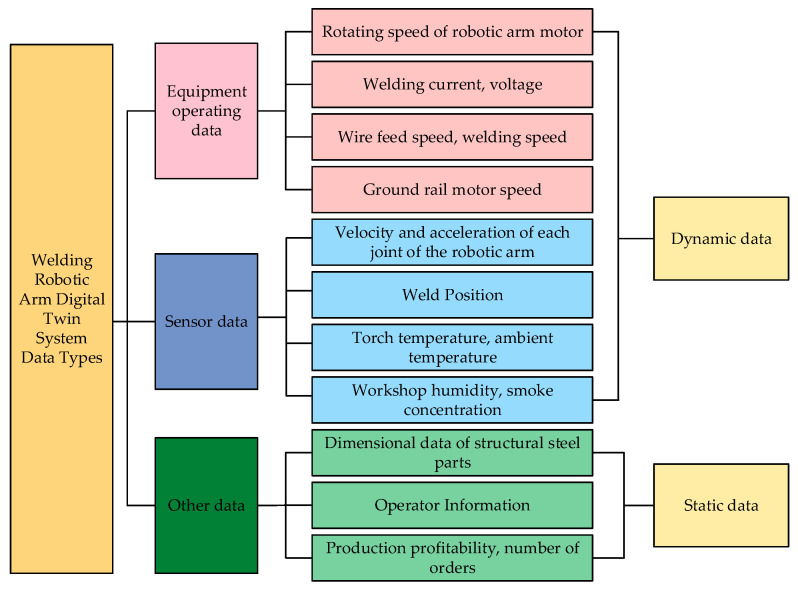
Data types of digital twin system for welding robotic arm.

By analyzing the workflow of the welding robot arm and combining the above requirements for data classification, the data information involved is divided into the following categories, as shown in Figure 25.

The welding process of “H” section steel components consists of the following steps: alignment, welding, and cooling. The use of different types of sensors ensures data acquisition for each step and visualization of the entire welding process. Position sensors are used to ensure that the “H” sections are correctly positioned before welding. Welding current, voltage, and temperature are monitored during the welding process. Current and voltage sensors are used to ensure that the welding parameters are within the set range, and temperature sensors are used to monitor the temperature of the weld point. In addition, temperature sensors are used to monitor the cooling rate. In terms of weld quality monitoring, the company currently uses manual ultrasonic flaw detection, so no sensors are placed in this area. Environmental sensors are also arranged throughout the whole workshop. Temperature and humidity sensors are used to ensure the welding environment is stable and prevent humidity from affecting the welding quality. Smoke sensors and flame sensors are used to prevent fires, protect personnel, and ensure equipment safety. Specific information is shown in Table 5.

**Figure 26:**
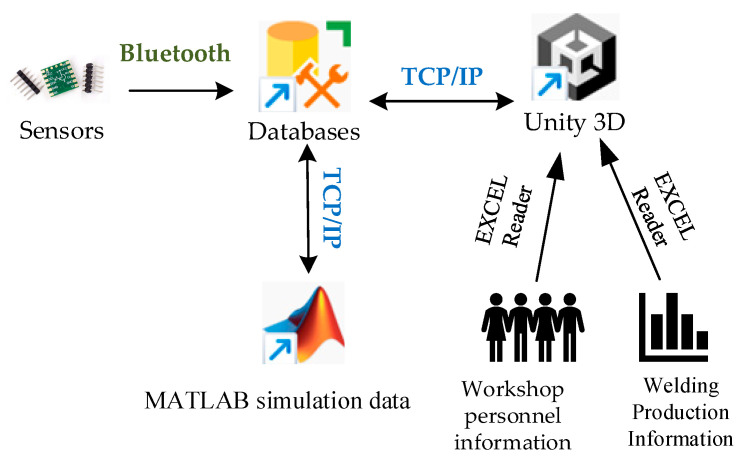
Data transmission route and communication mode.

The data transmission routes and communication methods are shown in Figure 26. The inclusion of data transmission between MATLAB and Unity3D in the data transmission network is because MATLAB serves as the platform for path planning of the welding robotic arm, and the path-planning data is directly simulated through Unity3D. This approach avoids the loss of manpower and financial resources that would otherwise occur when testing path planning on actual equipment.

In the development of the welding robotic arm digital twin system, Unity 3D, as a lightweight 3D development engine, can only be used as a visualization platform and does not have data storage capabilities. Therefore, during data collection and transmission, an intermediate medium is required for organizing, storing, and exchanging data. This medium is the database. Database systems support the storage of data types that fall into two broad categories: structured data and unstructured data. Database types in back-end development can be categorized into two types: relational and non-relational databases. All the data involved in the welding robot arm digital twin system is structured data, i.e., data stored in tabular form. Therefore, a relational database is chosen when selecting a database. Common database software includes the following: the Oracle database, MongoDB database, MySQL database, DB2 database, and SQL Server database. Considering the data type, the database needs to support data transfer with Unity 3D and MATLAB, so we finally choose the SQL Server database that can satisfy all data transfer [16].

In terms of data transmission technology, we combine TCP/IP with Bluetooth communication. This is because parallel transmission of multi-source data is faster and has a higher data integration rate. It enhances real-time monitoring and dynamic response capabilities, enabling synchronous collection of multidimensional data and rapid alerts for abnormal conditions, such as synchronous monitoring of current and welding gun temperature during welding, thereby reducing the rate of missed equipment abnormalities. It optimizes production efficiency and process stability, and supports dynamic optimization of welding parameters and coordinated scheduling of multiple devices, improving equipment operational efficiency. Additionally, it enhances system scalability and cost control capabilities, enabling flexible integration of heterogeneous devices and data-driven predictive maintenance, thereby reducing equipment integration and maintenance costs.

(1) TCP/IP Communication Protocol

**Figure 27:**
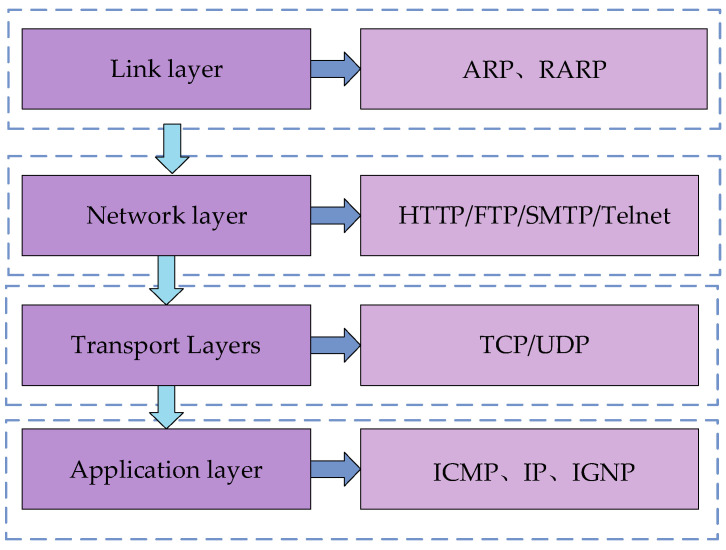
TCP/IP communication model.

The TCP/IP (Transmission Control Protocol/Internet Protocol) communication protocol is the basis for communication on the Internet and most LANs. The TCP/IP communication model consists of four layers: the application layer, transport layer, network layer, and link layer. The various protocols in the TCP/IP model are divided into these four layers according to the functions they fulfill. The transmission and processing of data are then realized through the interfaces of the two adjacent layers. As shown in Figure 27, the model of the TCP/IP communication protocol and the protocols contained in each layer are illustrated. The application layer, which is built on top of TCP or UDP, is used to define how applications exchange data and communicate; the reliability of the data transmission and whether the data reaches the receiving layer in the correct order is ensured by the transport layer. The IP address of the destination determines the next path of the transmitted data, which is then forwarded by the network layer. A hardware device—a router—is involved in this process. It can determine the transmission path based on the IP address and then transmit the data to the receiving host through the link layer, thus enabling the transmission of packets across different networks. The link layer refers to the hardware that handles the connection to the network.

The TCP header format contains key information used to establish, maintain, and terminate connections as well as to ensure the reliability of TCP communications.

**Figure 28:**
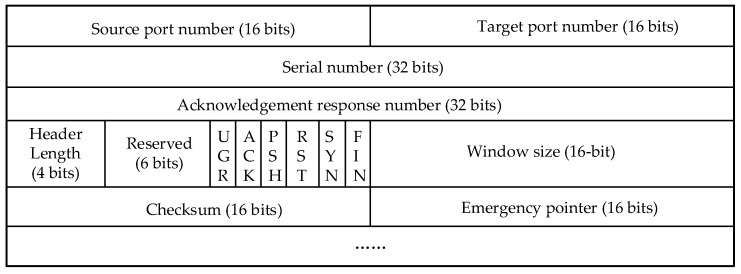
TCP header format.

Port numbers, serial numbers, acknowledgement numbers, control flags, and window sizes are used to ensure reliable data transmission, traffic control, and connection management. Check digits are used for error detection, and emergency pointers are used to handle emergencies. Together, this information ensures a clear connection orientation, and the TCP header format is shown in Figure 28.

The realization of the TCP protocol communication process includes “three handshakes” and “four handshakes”. The former is used to establish a connection, and the latter is used to terminate it. The steps to establish a connection are as follows:

(1) The first handshake: The client will send a special TCP segment to the server. This TCP segment not only carries the client that identifies the beginning of the data flow to the beginning of the sequence number (INS), but also initiates a request for the SYN flag bit, that is, the synchronization flag bit. The initial sequence number is seq = x, where x is a randomly generated value to ensure that the sequence number of each connection is unique, thus avoiding duplication or confusion of old connections.

(2) Second handshake: When the server receives the SYN segment from the client, if it decides to accept the connection request, it responds with a TCP segment that has both the SYN and ACK flag bits set. This response segment not only contains the server’s own initial sequence number (ISN), which identifies the starting point of its data stream, but also acknowledges the ISN previously sent by the client, i.e., ACK = x + 1, indicating the next sequence number expected to be received. In this segment, the SYN flag bit is likewise set to 1, indicating that the server is also in a synchronized state, while seq=y is the initial sequence number randomly generated by the server, ensuring the uniqueness and security of the connection.

(3) Third handshake: When the client receives the SYN-ACK segment from the server, it sends a TCP segment with the ACK flag bit as a final confirmation. This segment contains an acknowledgement of the server’s sequence number (ACK=y+1), indicating that the client has successfully received the server’s initial sequence number and is ready to start receiving data. At this point, the three-handshake process of the TCP connection is complete, and the two parties have established a reliable communication channel and can begin formal data transmission.

**Figure 29:**
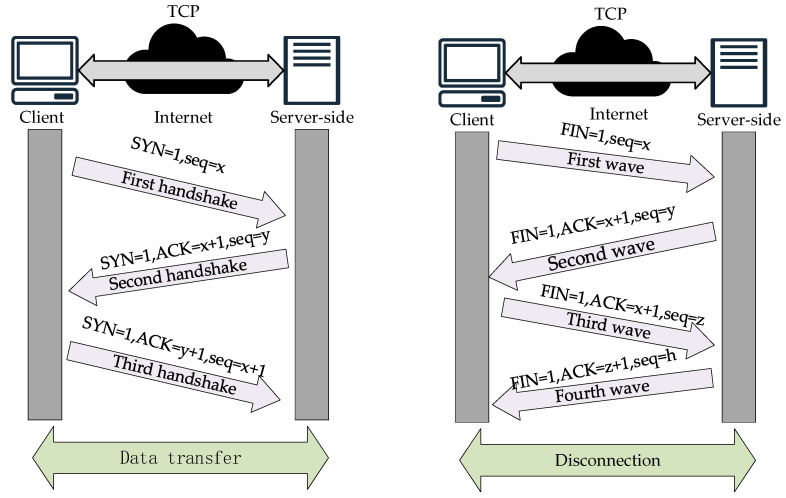
TCP communication protocol: three handshakes and four waves.

Since TCP connections are full-duplex, i.e., data can be transmitted in both directions at the same time, each direction needs to be closed independently when disconnecting. This bidirectional closure mechanism results in a four-wave process for disconnecting, as shown in Figure 29.

(1) First wave: The client indicates completion of data transfer and decides to close the connection when it sends a TCP segment with a FIN flag bit (end flag) to the server.

(2) Second wave: When the server receives the TCP segment with the FIN segment sent by the client, it then responds with a TCP segment with the ACK flag bit, indicating that it has confirmed that the client is ready to close the connection request.

(3) Third wave: When the client completes all data transmission, it sends a TCP segment with the FIN flag bit to the server, indicating that all data transmission from the client has been completed.

(4) Fourth wave: When the server receives the FIN segment sent by the client, it sends the last TCP segment with an ACK segment and then closes the connection. The client will also close the connection immediately after receiving the last TCP segment. At this point, the “four waves” are completed, and TCP communication is completely terminated.

(2) Bluetooth communication

When collecting sensor data information, the Bluetooth module is selected to send the data information to the upper computer database. Similar to the TCP/IP communication protocol, the implementation of Bluetooth communication relies on the Bluetooth protocol stack (BPS). This protocol stack specifies how data transfer and communication between Bluetooth devices take place.

**Figure 30:**
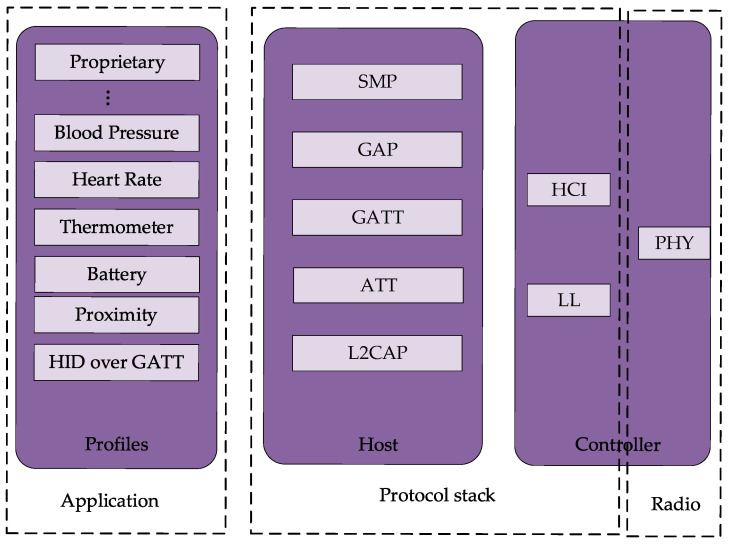
Bluetooth protocol stack structure.

The Bluetooth protocol stack consists of multiple layers, each of which bears different functions and responsibilities, and the specific hierarchical structure and the contents contained in each layer are shown in Figure 30 [17,18]. The application layer, as the top layer of the Bluetooth protocol stack, interacts directly with the application program interface (API) and provides functions such as file transfer, audio transmission, etc. The physical layer, as the bottom layer of the Bluetooth protocol stack, is responsible for radio-frequency data transmission by defining the wireless transmission rate, frequency, etc. The link layer is built between the application layer and the physical layer. The physical layer is the bottom layer of the Bluetooth stack, which is responsible for radio-frequency data transmission by defining the radio transmission rate, frequency, etc. The link layer is established between the application layer and the physical layer, which is responsible for transmitting and receiving data packets between layers. In addition, it also contains error detection and correction mechanisms, which can segment and reorganize the packets. The HC-05 Bluetooth module was finally selected by synthesizing the field equipment. This Bluetooth module is suitable for remote control, data logging applications, robotics, and monitoring systems. Based on the Bluetooth 2.0 protocol, it is able to support data transmission between two microcontrollers with serial communication capability, as well as control and manage other Bluetooth devices through the microcontroller.

(3) ExcelDataReader Library

Workshop operators, shift conditions, order profitability, and other related data are stored directly in the form of Excel tables in the host computer, so one can write C# scripts in Unity 3D to call the ExcelDataReader library to read Excel table information. The ExcelDataReader library is used to read Excel files, and it is open source. The NET library supports reading a variety of Excel file formats. It also provides a simple API to extract data from Excel files. Welding robot arm data stored in the Excel table format is in “.xls” format.

### 4.3. Validation of Data Transmission Results

(1) Data transfer between sensors and database

**Figure 31:**
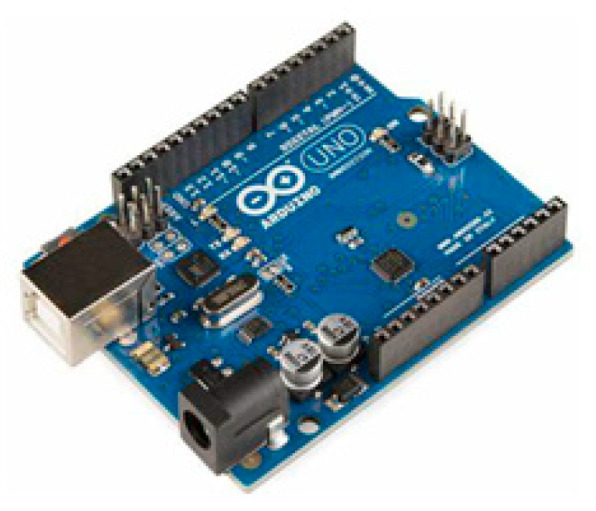
Arduino Uno development board.

**Figure 32:**
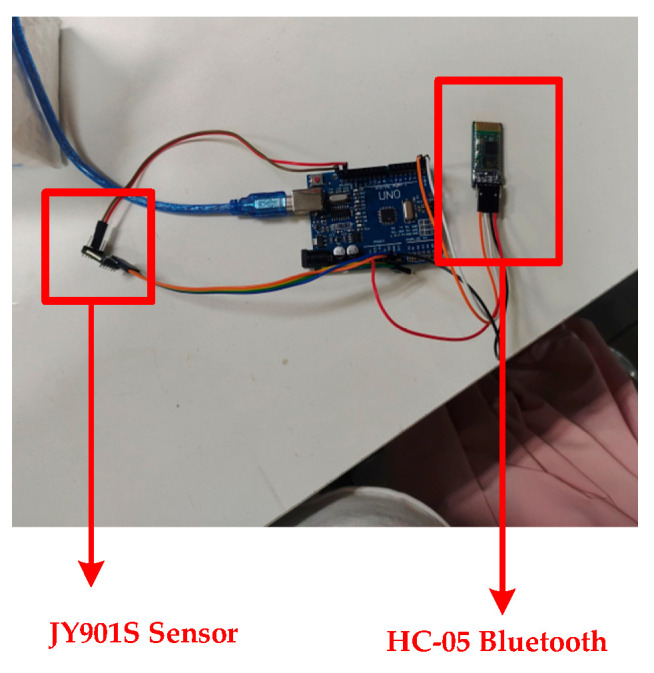
Sensor information acquisition equipment.

Take the JY901S sensor as an example to introduce the sensor and the data transmission in the database. The sensor is placed at the end of the robotic arm actuator, responsible for the collection of the angle at the end of the robotic arm execution, angular acceleration, and so on. Before the data collection, it is necessary to connect the sensor with the Bluetooth module with the help of an Arduino Uno development board, as shown in Figure 31 and Figure 32. Since the sensor cannot transmit the data to the PC alone, it is necessary to use a microcontroller for signal processing and converting the sensor’s signal into a digital signal. In addition, the microcontroller can control the Bluetooth module to send data. After the Bluetooth module receives the digital signal, the digital information is written into the SQL Server database remotely with the help of the Bluetooth communication protocol. Thus, data transfer between the sensor and the database is achieved.

**Figure 33:**
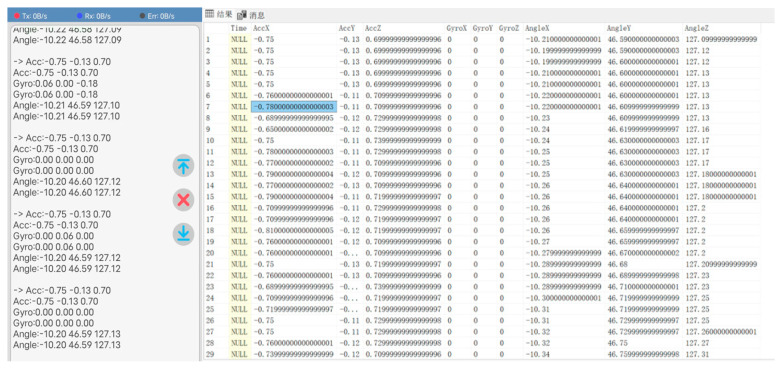
Sensor-received data written to SQL database.

The collected JY901S sensor angle data and acceleration data are shown in Figure 33 (left), and the sensor data obtained from the mobile end is stored in the corresponding SQL Server database table. The storage result is shown in Figure 33 (right).

(2) Data transfer between MATLAB and database

**Figure 34:**
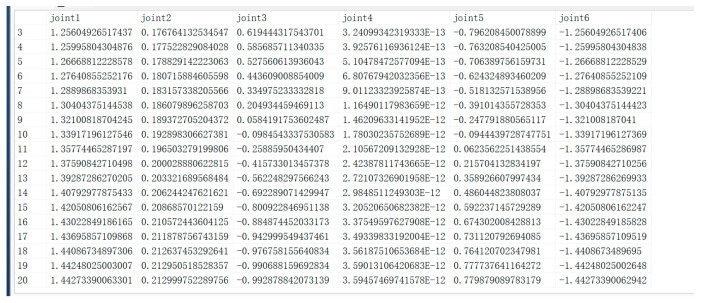
Data transfer results.

The configuration of the development environment between MATLAB and the database needs to be established before data transfer, i.e., establishing ODBC (Open Database Connectivity) communication. ODBC (Open Database Connectivity) is a standardized interface. Its main use is to establish communication between the database and the application program. Because it allows applications to access various databases using SQL statements, it is no longer necessary to write specific code programs for each database. ODBC simplifies the cumbersome process of database–application interaction by providing an interface layer under a unified standard. After completing the configuration of the development environment, we tested the data transfer function. The output of the algorithm is the path points after path planning, and these path points are written in the “JointAngle” table in the SQL Server database. Each time the algorithm recalculates the path points, the data in the database needs to be synchronized and updated to ensure that the latest path point information can be uploaded and replace the old data in time. This process not only requires an efficient data transfer mechanism but also must ensure the consistency and accuracy of the data, so as to provide a reliable basis for subsequent operations. The results of data transmission are shown in Figure 34.

(3) Data transfer between Unity 3D and the database

**Figure 35:**
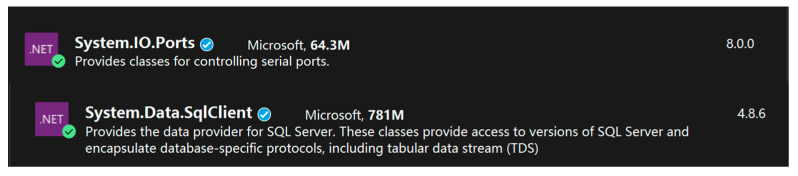
Extended procedure set.

Before realizing the data transfer between Unity 3D and the database, the environment of the software must be configured at both ends. Unity 3D realizes the data transfer between Unity 3D and the database with the help of Visual Studio programming software and the C# language. Unity 3D software needs to configure the I18N.CJK.dll, I18N.dll, I18N.West.dll, and System.Data.dll files of the project in the Plugins file. CJK.dll, I18N.dll, and I18N.West.dll are used by Unity 3D to handle text display, character encoding, sorting, etc. System.Data.dll ensures that Unity 3D accesses the call data. The Visual Studio side needs to be configured with System.IO.Ports and System.Data.SqlClient, as shown in Figure 35. The System.IO.Ports assembly provides developers with the ability to manipulate serial ports, enabling them to transfer and control data with hardware devices through serial communication. The System.Data.SqlClient assembly is primarily used to interact with Microsoft SQL Server databases. SqlClient defines some functions to help users connect to SQL Server databases, as well as perform data queries, calls, and so on.

Next, the TCP/IP communication protocol is enabled in the SQL Server Database Configuration Manager to ensure that the database can communicate over the network.

**Figure 36:**
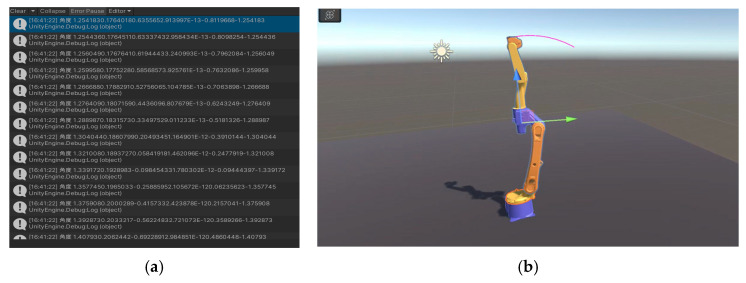
Unity 3D and MATLAB data interaction and trajectory replication. (**a**) Unity 3D reads MATLAB data. (**b**) Replication of circular trajectories in MATLAB in Unity 3D.

After the configuration of the environment files required above is completed, a corresponding C# script is written to control the access and invocation of the SQL Server database information by Unity 3D. A small section of the path planned by the circular interpolation of the robotic arm was selected in this test phase, and the result of reading data is shown in Figure 36a. The script controls the robotic arm to assign the read rotation angles of each joint to reproduce the trajectory of the robotic arm, and the result of the reproduced trajectory is shown in Figure 36b.

(4) Unity 3D reading Excel table information

**Figure 37:**
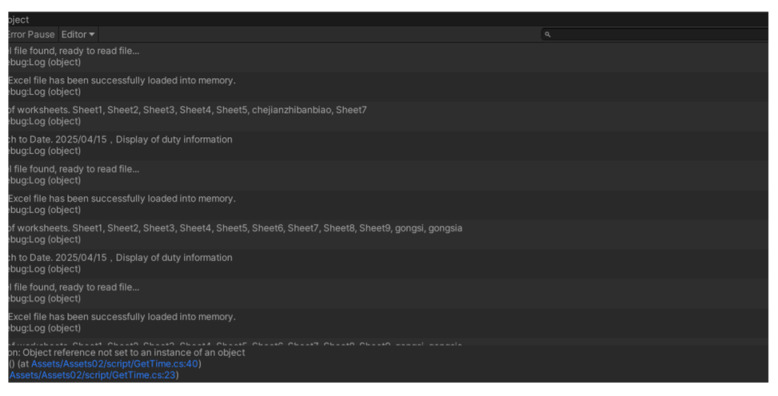
Unity 3D establishes a connection to an Excel file.

The library file in Unity 3D is configured, and the ExcelDataReader.dll and ExcelDataReader.DataSet.dll files are imported into the Plugins file. Then, a C# script is written to realize the reading of the corresponding table content. The process of reading data after successful connection is shown in Figure 37. Data visualization will be described in detail in the UI section later in this chapter.

(5) Synchronized validation of Unity 3D side with physical side

**Figure 38:**
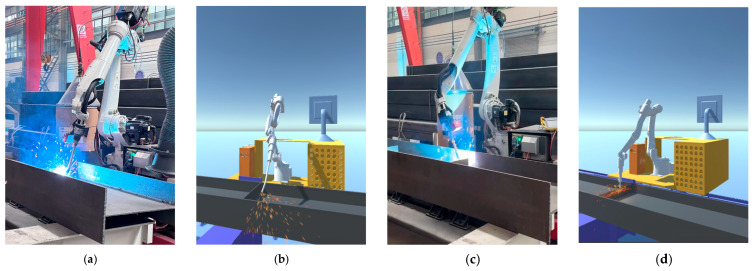
Synchronized verification results between Unity 3D side and physical side. (**a**) Entity end in posture (1). (**b**) Virtual end in posture (1). (**c**) Entity end in posture (2). (**d**) Virtual end in posture (2).

In the previous section, the JY901S sensor was used to complete the data acquisition of the end-effector of the welding robot arm, and it was confirmed that the data can be properly transferred into the SQL Server database. It also completed the reading and writing of data between Unity 3D and the database. Now, an inverse kinematic function script is written in Unity 3D using C# to calculate the joint angle of the end-effector to reach the target position and attitude according to the position and attitude of the welding robot arm end-effector. Then, according to the calculation results, the welding robot arm joints are controlled to rotate to the specified angle to achieve synchronization with the physical end of the welding robot arm. The verification results are shown in Figure 38.

## 5. UI Design for Digital Twin System for Welding Robot Arm

The advantage of the system is that the various types of complex algorithms involved can be integrated and encapsulated as a whole, and buttons and other functions can be used to realize the rapid invocation of each algorithm. The preliminary data communication work is completed with the help of the Unity 3D development engine and the C# language to develop the welding robot arm digital twin system. The whole system interface includes three parts: login interface, data interaction and visualization interface, and simulation interface. Realizable functions include 3D visualization of the welding model, real-time monitoring of field operation, stopping the robotic arm operation in case of emergencies, and simulating the running path of the robotic arm.

### 5.1. Login Interface Design

The purpose of designing the login interface is mainly to enhance the privacy of the system and avoid the leakage of internal company data. All the system’s service functions are integrated with the identity authentication to ensure that only internal employees are allowed to access the system resources and operate the system through an encrypted channel.

**Figure 39:**
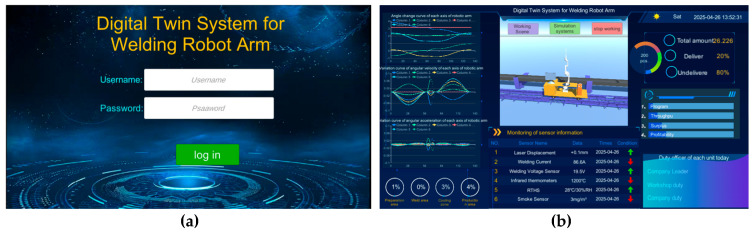
(**a**) System login screen. (**b**) Data visualization interface.

As shown in Figure 39a above, the Text-Input Field component and button component in the UI system in Unity 3D are utilized to complete the transformation of the system scene, and after entering the user’s name (AAA) as well as the password (111), clicking the login button allows the user to enter the data visualization interface of the system, as shown in Figure 39b. All functions and services of the system are open to users.

### 5.2. Data Visualization Interface Design

The construction of the UI of the welding robotic arm digital twin system adopts a modular design, with different functions performed by different modules. This design is inspired by the different functions performed by different modules, and the construction of the UI utilizes the multi-scene asynchronous loading technology of the Unity 3D engine, which breaks down the core functions of the system into two independently running units: the data interaction scene and the simulation scene. This modular design not only effectively handles and visualizes complex multi-source data but also avoids the risk of resource hogging in the traditional single-scene mode.

**Figure 40:**
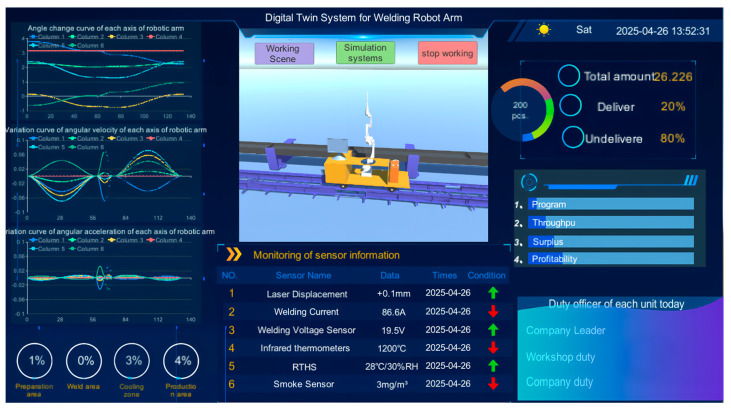
System data visualization interface.

The system data visualization interface adopts a responsive UI design, and the overall layout is shown in Figure 40. The monitoring matrix, built based on Canvas components, contains five major functional blocks. The 3D visualization dashboard supports LOD hierarchical rendering for 3D model rotation display; the data curve monitoring area feeds back the kinematic data of the robotic arm joints. The business status dashboard circularly displays the order delivery situation, and the bar chart shows the order profitability trend; the sensor array feeds back the on-site environment and welding situation; the personnel management panel displays the current information of the person in charge of each area, and the responsibility is fine-tuned for responding to unexpected situations.

The system re-reads database data every 5 s, with new data overwriting the previous data. By measuring the synchronization delay between system data and actual data, the delay is kept within 1 s, meeting the delay time requirements for the current system functionality. In the future, if the system needs to add multiple robot arms of the same model, you can simply copy them from the model library. As for adding more sensors, you must first transfer the sensors to the database via Bluetooth. After that, add programming statements in Unity 3D to read the corresponding position data.

**Figure 41:**
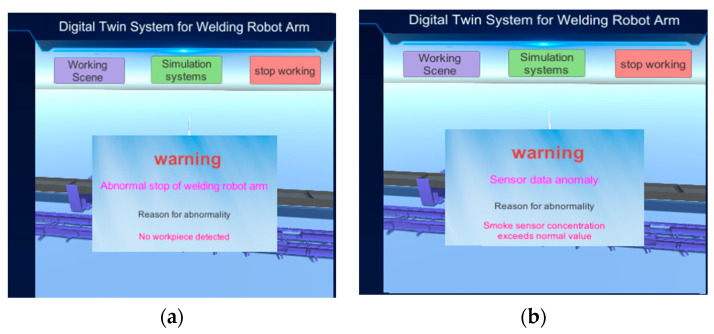
System warning screen. (**a**) Welding robot abnormal shutdown warning. (**b**) Sensor abnormality warning.

In addition, the C# programming language is used to continuously monitor the acquired real-time data. If any abnormal conditions are detected in the monitored data, a warning is immediately displayed on the system interface, explaining the abnormal condition and its cause. As shown in Figure 41, (a) the warning indicates an abnormal stop of the robotic arm, with the cause being that the workpiece position sensor did not detect the workpiece; (b) the warning indicates a sensor abnormality, with the cause being that the smoke sensor data exceeded the normal range.

In addition to the above main functions, two button components are added to the model display area, giving the component the function of calling the scene conversion script, which jumps to the working scene of the robotic arm and the simulation scene, respectively, after clicking.

### 5.3. Work Environment Module Design

New employees or technicians familiarizing themselves with the complex welding scene and the operation process of the robot arm need a lot of time and practice. The development of an immersive virtual scene interaction module can quickly solve the above problems. The module includes the following functions: scene roaming, equipment introduction, workflow introduction, and welding process simulation.

**Figure 42:**
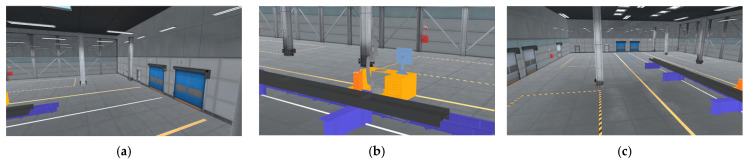
Roaming view. (**a**) Roaming down perspective I. (**b**) Roaming down perspective II. (**c**) Roaming down perspective III.

During the roaming process, the main camera in the Unity 3D scene is controlled by the mouse, as shown in Figure 42. Horizontal movement of the mouse controls the left and right rotation of the camera, vertical movement controls the up and down rotation, and the up and down rotation angle is limited to between -90° and 90° to prevent excessive rotation. The mouse wheel is responsible for adjusting the distance to the target point, and the zoom distance is limited to [minZoomDistance, maxZoomDistance], where minZoomDistance=1f (i.e., 1 meter) and maxZoomDistance=10f (i.e., 10 m), and utilizes Unity 3D’s LateUpdate callback method in Unity 3D to ensure that the camera rotation and position are updated after all other objects have been updated, thus avoiding camera shake.

**Figure 43:**
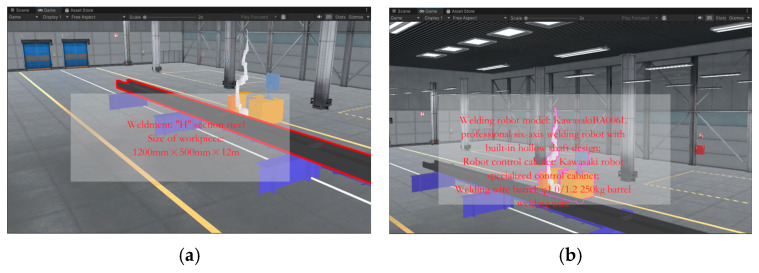

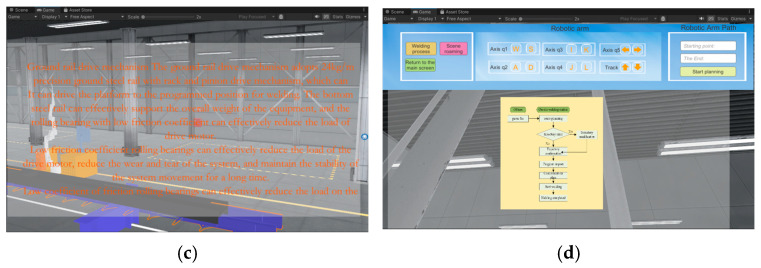
Equipment and workflow introduction. (**a**) Weldment size information. (**b**) Introduction to welding consoles. (**c**) Ground track—introduction to the seventh axis. (**d**) Introduction to the welding process.

The device introduction and workflow introduction are realized by clicking on the object, as shown in Figure 43. When the object senses the clicked input in the interaction interface, it can enable the corresponding panel, and the system’s perception of user input relies on the ray collision detection technology in the Unity 3D engine, which is realized by the Physics Recaster in the Event System. When mounted on the mouse, it emits an infinitely long ray of light from the mouse in a certain direction and monitors the ray path in real time for input events such as clicks and selections. It then passes this input information to a C# script that triggers the model to respond with the appropriate output. It is important to note that every object that needs to be clicked to trigger an effect should have a collision body added; otherwise, the ray will pass right through the object and will not be recognized.

### 5.4. Motion Simulation Module and Path-Planning Design

The motion simulation in this system is a technical means to control the rotation of the joints of the robotic arm through the script, which can highly realistically restore the operation process of the welding robotic arm, in order to further enhance the authenticity while adding a particle welding effect. The simulation module allows users to quickly and intuitively understand the structure of the arm, the motion characteristics of the joints, and the complete welding process.

**Figure 44:**
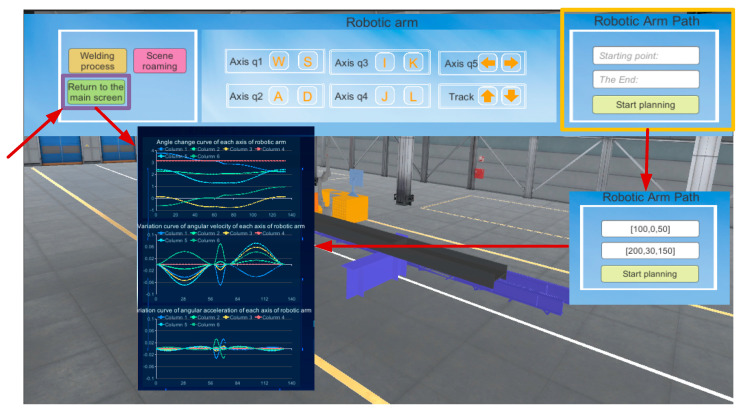
Motion simulation module and path planning.

**Figure 45:**
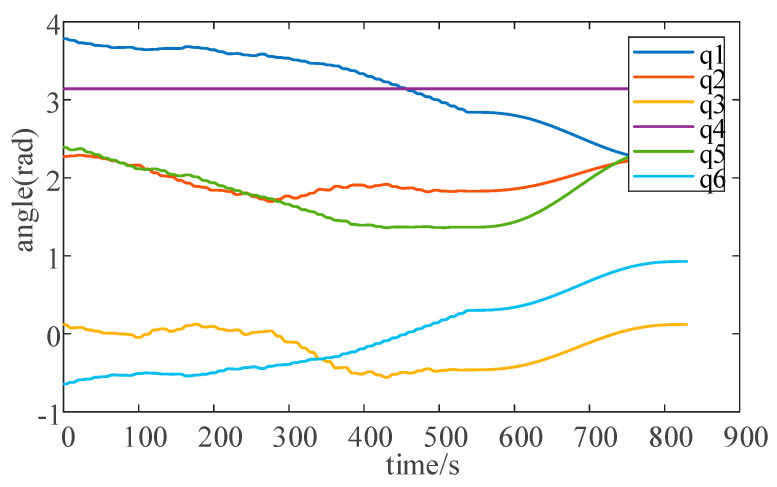
Angle curves of each axis when the robotic arm follows the planned path.

**Figure 46:**
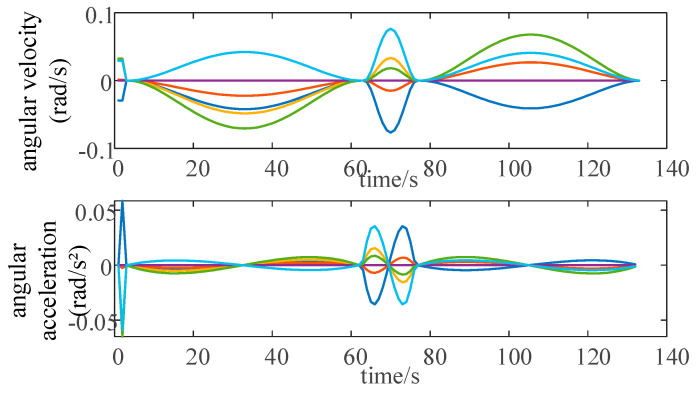
Angular velocity and angular acceleration curves of each axis of the robotic arm along the planned path.

The corresponding buttons assigned to each joint of the robot arm in the system interface are shown in Figure 44. The user can precisely operate the joints of the robot arm in this way. The upper right side of the figure is the robotic arm path-planning module, which mainly utilizes the Text-Input Field component to complete the input of the starting point and end point of the path, and is controlled by the C# script to input the data into the path-planning algorithm. With the help of the button component, it invokes the MATLAB file and ultimately realizes the path planning of the two target points. In MATLAB, the path planning for the robotic arm is implemented using the RRT algorithm. Figure 45 shows the angle curves of each axis as the robotic arm follows the planned path, while Figure 46 displays the angular velocity and angular acceleration curves of each axis as the robotic arm follows the planned path. After the planned path is completed, the results of the movement of each joint of the robotic arm will be fed back to the system’s data visualization interface in real time, and the user can return to the data visualization interface at any time to check. When ensuring that the planned path meets the requirements of practical applications, the robot arm path point file is saved for offline verification.

### 5.5. Maturity Assessment of Digital Twins for Welding Robotic Arms

**Figure 47:**
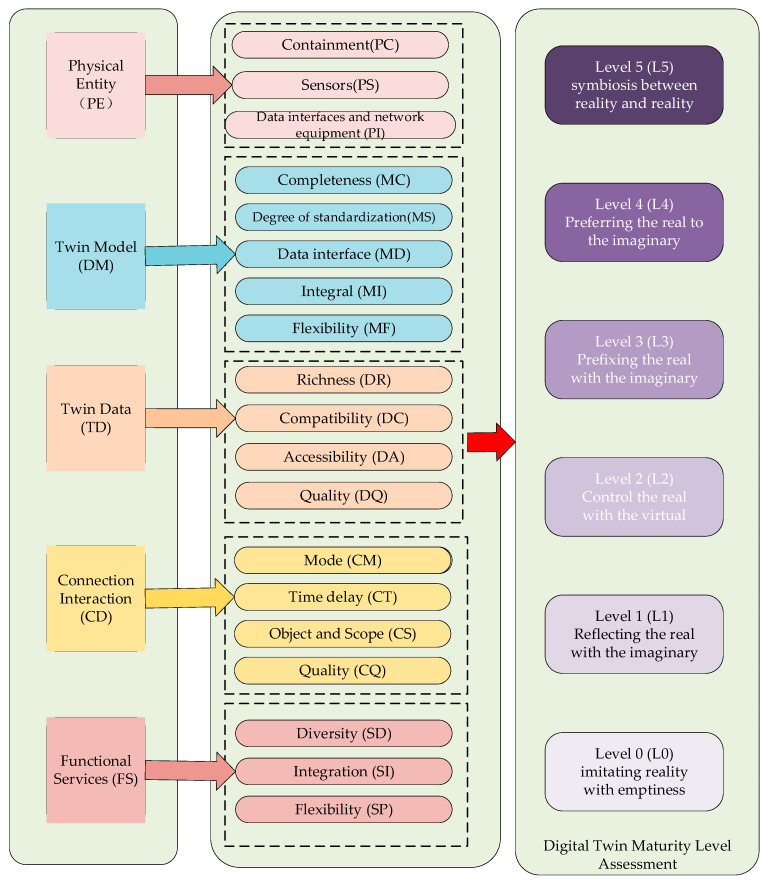
Digital twin maturity model framework diagram.

The digital twin maturity model was proposed by Prof. Tao Fei et al. to evaluate the maturity of digital twins, and it provides clear guidance for the evaluation and optimization of digital twin systems [19]. The welding robotic arm digital twin maturity model consists of two main parts: digital twin maturity level and evaluation factors. The maturity level clearly describes the functions and characteristics of the digital twin system at different stages of development and is the core part of evaluating the digital twin system. The evaluation factors provide a more detailed assessment of the digital twin in five dimensions. These five dimensions are the physical entity (PE), twin model (DM), twin data (DD), connectivity interaction (CI), and service functionality (FS). Each dimension is further subdivided into 19 specific evaluation metrics. The specific compositional framework of the digital twin maturity model is shown in Figure 47.

The maturity level of each evaluation factor is determined in conjunction with the current welding robotic arm digital twin system, as shown in Table 6, and then the total score is calculated based on the weights of each dimension to finalize the maturity level. Since the importance of each link in the digital twin system is the same, the weights involved in the entire calculation process are calculated using average weights.

The comprehensively calculated maturity evaluation score of the welding robotic arm digital twin system is 2.74, which meets the requirements of the second level of digital twin maturity, partly satisfies the control of the physical entity by the virtual model, and greatly satisfies the reflection of the physical entity by the virtual model. In general, there is still a lot of optimization space for this system. From the data in the radar chart of the evaluation factors in five dimensions, it can be seen that the physical entity of the welding robotic arm, the system connection interaction, and the functions and services have lower scores; from the view of the 19 refined evaluation factors, the evaluation factors of MF, CT, and SD are lower and need to be further improved, see Figure 8.

## 6. Conclusions

In conclusion, this study focuses on the construction of a digital twin model and system development for a welding robot arm, achieving a series of important results. The research utilizes software such as SolidWorks, 3dsMax, and Unity 3D to accurately draw a 3D model of the welding production line and perform lightweight processing, format conversion, and parent–child hierarchy construction. Through rendering, particle welding effects are created to enhance the model’s realism. Meanwhile, a data-driven digital twin system for the welding robot arm is successfully constructed. By leveraging communication protocols such as TCP and Bluetooth, data interaction is achieved between the physical and virtual ends, as well as between Unity 3D and MATLAB software. The UI function of the Unity 3D development engine is used to complete the system design, and the maturity of the constructed system is evaluated based on the digital twin system maturity model proposed by Tao Fei’s team. Test results show that integrating digital twin technology with automation in the field of welding robot arms is feasible and achieves the expected results. The application of digital twin technology to welding robotic arm workstations is a representative example of digital empowerment in the smart manufacturing industry. Its successful implementation in welding processes provides a referable example for the automation of other industrial processes.

However, limited by time and technical conditions, this study still has certain limitations. The currently developed digital twin system for welding robot arms is relatively limited in terms of functions and services, and there is significant room for improvement in the system’s final maturity score. Future research will focus on expanding system functions, optimizing data interaction and processing mechanisms, and further enhancing the system’s intelligence level and maturity to provide more comprehensive and efficient digital twin solutions for the intelligent manufacturing field.

## Figures and Tables

**Figure 48:**
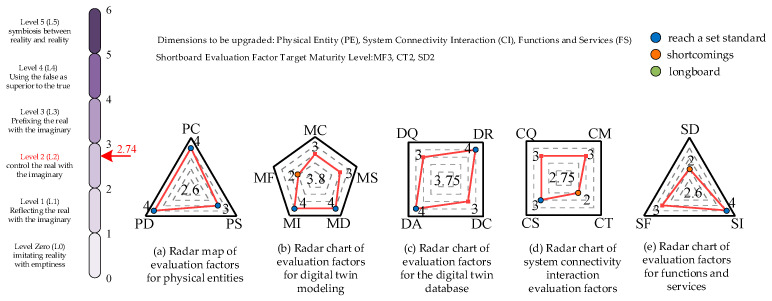
Welding robotic arm digital twin maturity assessment results.

**Table 1:** Rotation range angles of each joint of robotic arm.

Action Axis	Maximum Range of Motion
arm rotation (q1)	−165~+165°
arms front and back (q2)	−90~+150°
arms up and down (q3)	−175~+90°
arm rotation (q4)	−180~+180°
flexion of the wrist (q5)	−135~+135°
wrist twist (q6)	−360~+360°

**Table 2:** Optimization results for the number of vertices and faces of the model.

Equipment Name	Number of Vertices	Number of Vertices After Optimization	Number of Faces	Number After Optimization
Mechanical arm model	4001	1983	7699	3783
Operation table model	16,305	8148	32,926	16,612
Ground rail model	48,173	19,269	96,794	38,986
Welding support model	534	213	1072	430

**Table 3:** Particle effect key parameter settings.

Title	Parameters	Title	Parameters
Start Lifetime	2	Shape	Sphere
Start Size	0.1, 0.25	Radius	0.28
Start Color	FFA500	Size over Lifetime	10
Start Speed	4.5	Material	Default-ParticleSystem
Emission	200	Render Mode	Stretched Billboard
Light	Sparks_Light	Render Speed Scale	0.03

**Table 4:** Reference coordinate system and motion axis of each joint of robotic arm.

Title	Coordinate System of Reference	Axis of Motion	Movement Style
Joint q1	local coordinate system	Z-axis	axis of rotation
Joint q2	local coordinate system	X-axis	axis of rotation
Joint q3	local coordinate system	X-axis	axis of rotation
Joint q4	local coordinate system	Z-axis	axis of rotation
Joint q5	local coordinate system	X-axis	axis of rotation
Joint q6	local coordinate system	Z-axis	axis of rotation
Control panel	local coordinate system	X-axis	lateral movement

**Table 5:** Sensor-related information.

Sensor Name	Typology	Mounting Position	Monitoring Parameters	Normal Range
Laser displacement sensor	Photoelectric	Welding bracket	“H” section position deviation	±1 mm
Welding current sensor	Hall effect sensors	Torch power cord	Welding current	200–250 A
Welding voltage sensor	Voltage sensors	Welding gun controller	Welding voltage	20–30 V
Infrared thermometer	Non-contact thermal sensors	Welding torch neighborhood	Welding point temperature	1200–1500 °C
Ambient temperature and humidity sensors	Temperature and humidity sensors	Center of workshop	Temperature/humidity	20–30 °C/30–60% RH
Smoke sensor	Gas sensors	Top of welding area	Fume concentration	<5 mg/m^3^

**Table 6:** Maturity levels of evaluation factors for digital twin system of welding robotic arm.

Evaluation Factor	Maturity Rating	Evaluation Factor	Maturity Rating
PC	4	DR	4
PS	4	DC	3
PI	4	DA	4
MC	3	DQ	3
MS	3	CM	3
MD	4	CT	2
MI	4	CS	3
MF	2	CQ	4
SD	2	SI	4
SF	3		

## Data Availability

All data generated or analyzed during this study are included in this published article.
